# Supplementary material for: Maternal and child outcomes for pregnant women with pre-existing multiple long-term conditions: protocol for an observational study in the UK
Source: BMJ Open. 2023 Feb 24;13(2):e068718. doi: 10.1136/bmjopen-2022-068718 (PMC9972454; doi:10.1136/bmjopen-2022-068718)
Supplement: Author's manuscript [file bmjopen-2022-068718.draft_revisions.pdf]

BMJ Open

BMJ Open is committed to open peer review. As part of this commitment we make the peer review history of every article we publish publicly available.

When an article is published we post the peer reviewers' comments and the authors' responses online. We also post the versions of the paper that were used during peer review. These are the versions that the peer review comments apply to.

The versions of the paper that follow are the versions that were submitted during the peer review process. They are not the versions of record or the final published versions. They should not be cited or distributed as the published version of this manuscript.

BMJ Open is an open access journal and the full, final, typeset and author-corrected version of record of the manuscript is available on our site with no access controls, subscription charges or pay-per-view fees (http://bmjopen.bmj.com).

If you have any questions on BMJ Open's open peer review process please email info.bmjopen@bmj.com

Maternal and children's outcomes for pregnant women with pre-existing multiple long-term conditions: a study protocol of an observational study in the United Kingdom

Journal:	*BMJ Open*
Manuscript ID	bmjopen-2022-068718
Article Type:	Protocol
Date Submitted by the Author:	28-Sep-2022

Complete List of Authors:	Lee, Siang Ing; University of Birmingham Institute of Applied Health Research<br>Hope, Holly; The University of Manchester, Centre for Women's Mental Health, Faculty of Biology Medicine & Health<br>OReilly, Dermot; Queen's University Belfast, Centre for Public Health<br>Kent, Lisa; Queen's University Belfast, Centre for Public Health<br>Santorelli, Gillian; Bradford Institute for Health Research<br>Subramanian, Anuradhaa; University of Birmingham Institute of Applied Health Research<br>Moss, Ngawai; Patient and public representative<br>Azcoaga-Lorenzo, Amaya; University of St Andrews School of Medicine, Division of Population and Behavioural Sciences<br>Fagbamigbe, Adeniyi; University of St Andrews School of Medicine, Division of Population and Behavioural Sciences; University of Ibadan College of Medicine, Department of Epidemiology and Medical Statistics<br>Nelson-Piercy, Catherine; Guy's and St Thomas' NHS Foundation Trust<br>Yau, Christopher; University of Oxford, Nuffield Department of Women's and Reproductive Health; Health Data Research UK<br>McCowan, Colin ; University of St Andrews School of Medicine, Division of Population and Behavioural Sciences<br>Kennedy, Jonathan; Swansea University, Data Science, Medical School<br>Phillips, Katherine; University of Birmingham Institute of Applied Health Research<br>Singh, Megha; University of Birmingham Institute of Applied Health Research<br>Mhereeg, Mohamed; Swansea University, Data Science, Medical School<br>Cockburn, Neil; University of Birmingham Institute of Applied Health Research<br>Brocklehurst, Peter; University of Birmingham Institute of Applied Health Research<br>Plachcinski, Rachel; Patient and public representative<br>Riley, Richard; Keele University School of Primary Community and Social Care, Centre for Prognosis Research<br>Thangaratinam, Shakila; University of Birmingham Institute of Metabolism and Systems Research, WHO Collaborating Centre for Global Women's Health; Birmingham Women's and Children's NHS Foundation Trust, Department of Obstetrics and Gynaecology<br>Brophy, Sinead; Swansea University, Data Science, Medical School<br>Hemali Sudasinghe, Sudasing Pathirannehelage Buddhika; University of

	Birmingham Institute of Applied Health Research<br>Agrawal, Utkarsh; University of St Andrews School of Medicine, Division of Population and Behavioural Sciences<br>Vowles, Zoe; Guy's and St Thomas' NHS Foundation Trust<br>Abel, Kathryn ; The University of Manchester, Centre for Women's Mental Health, Faculty of Biology Medicine & Health; Greater Manchester Mental Health NHS Foundation Trust<br>Nirantharakumar, Krishnarajah; University of Birmingham Institute of Applied Health Research<br>Black, Mairead; University of Aberdeen School of Medicine Medical Sciences and Nutrition, Aberdeen Centre for Women's Health Research<br>Eastwood, Kelly-Ann; Queen's University Belfast, Centre for Public Health; University Hospitals Bristol and Weston NHS Foundation Trust<br>-, on behalf of MuM-PreDiCT; MuM-PreDiCT
Keywords:	Maternal medicine < OBSTETRICS, OBSTETRICS, EPIDEMIOLOGY

SCHOLARONE™
Manuscripts

Title: Maternal and children's outcomes for pregnant women with pre-existing multiple long-term conditions: a study protocol of an observational study in the United Kingdom

Authors:

Siang Ing Lee,[1] Holly Hope,[2] Dermot O'Reilly,[3] Lisa Kent,[3] Gillian Santorelli,[4] Anuradhaa Subramanian,[1] Ngawai Moss,[5] Amaya Azcoaga-Lorenzo,[6] Adeniyi Francis Fagbamigbe,[6, 7] Catherine Nelson-Piercy,[8] Christopher Yau,[9,10] Colin McCowan,[6] Jonathan I Kennedy,[11] Katherine Phillips,[1] Megha Singh,[1] Mohamed Mhereeg,[11] Neil Cockburn,[1] Peter Brocklehurst,[1] Rachel Plachcinski,[5] Richard Riley,[12] Shakila Thangaratinam,[13, 14] Sinead Brophy,[11] Sudasing Pathirannehelage Buddhika Hemali Sudasinghe,[1] Utkarsh Agrawal,[6] Zoe Vowles,[8] Kathryn M Abel,[2, 15] * Krishnarajah Nirantharakumar,[1]* Mairead Black,[16]* Kelly-Ann Eastwood,[3, 17]* on behalf of the MuM-PreDiCT Group.

*Joint senior authors

Corresponding author: Krishnarajah Nirantharakumar, K.Nirantharan@bham.ac.uk

Affiliation:

1. Institute of Applied Health Research, University of Birmingham, Birmingham, UK
2. Centre for Women's Mental Health, Faculty of Biology Medicine & Health, The University of Manchester, UK
3. Centre for Public Health, Queen's University of Belfast, UK
4. Bradford Institute for Health Research, UK
5. Patient and public representative, UK
6. Division of Population and Behavioural Sciences, School of Medicine, University of St Andrews, UK
7. Department of Epidemiology and Medical Statistics, College of Medicine, University of Ibadan, Nigeria
8. Guy's and St. Thomas' NHS Foundation Trust, UK
9. Nuffield Department of Women's and Reproductive Health, University of Oxford, UK
10. Health Data Research UK
11. Data Science, Medical School, Swansea University, UK
12. Centre for Prognosis Research, School of Primary, Community and Social Care, Keele University, UK

For peer review only - http://bmjopen.bmj.com/site/about/guidelines.xhtml

13. WHO Collaborating Centre for Global Women's Health, Institute of Metabolism and Systems Research, University of Birmingham, UK
14. Department of Obstetrics and Gynaecology, Birmingham Women's and Children's NHS Foundation Trust, UK
15. Greater Manchester Mental Health NHS Foundation Trust, Manchester, UK
16. Aberdeen Centre for Women's Health Research, School of Medicine, Medical Science and Nutrition, University of Aberdeen, UK
17. St Michael's Hospital, University Hospitals Bristol NHS Foundation Trust, UK

Authors' ORCID:

Siang Ing Lee 0000-0002-2332-5452, Holly Hope 0000-0002-4834-6719, Dermot O'Reilly 0000-0002-9181-0652, Lisa Kent 0000-0002-8882-0526, Gillian Santorelli 0000-0003-0427-1783, Anuradhaa Subramanian 0000-0001-8875-7363, Ngawai Moss 0000-0001-9369-5072, Amaya Azcoaga-Lorenzo 0000-0003-3307-878X, Adeniyi Francis Fagbamigbe 0000-0001-9184-8258, Catherine Nelson-Piercy 0000-0001-9311-1196, Christopher Yau 0000-0001-7615-8523, Colin McCowan 0000-0002-9466-833X, Jonathan I Kennedy 0000-0002-1122-6502, Katherine Phillips 0000-0003-0674-605X, Megha Singh 0000-0003-3680-7124, Mohamed Mhereeg 0000-0003-1241-9549, Neil Cockburn 0000-0001-9284-6991, Peter Brocklehurst 0000-0002-9950-6751, Rachel Plachcinski 0000-0001-9908-0773, Richard Riley 0000-0001-8699-0735, Shakila Thangaratinam 0000-0002-4254-460X, Sinead Brophy 0000-0001-7417-2858, Utkarsh Agrawal 0000-0001-5181-6120, Zoe Vowles 0000-0001-6989-2180, Kathryn M Abel 0000-0003-3538-8896, Krishnarajah Nirantharakumar 0000-0002-6816-1279, Mairead Black 0000-0002-6841-8601, Kelly-Ann Eastwood 0000-0003-3689-0490.

Main text word count: 3119

Keywords: multimorbidity, multiple chronic conditions, multiple long-term conditions, pregnancy, maternity, obstetric, outcome, children, offspring

For peer review only - http://bmjopen.bmj.com/site/about/guidelines.xhtml

ABSTRACT

Introduction: One in five pregnant women have multiple long-term conditions in the United Kingdom (UK). Studies have shown that maternal multiple long-term conditions are associated with adverse outcomes. This observational study aims to compare maternal and children's outcome for pregnant women with multiple long-term to those without multiple long-term conditions.

Methods and analysis: Pregnant women aged 15 to 49 years old with a conception date between 2000 and 2019 in the UK will be included. The data source will be routine health records from all four UK nations (Clinical Practice Research Datalink [CPRD, England], Secure Anonymised Information Linkage [SAIL, Wales], Scotland routine health records and Northern Ireland Maternity System [NIMATS]), and the Born in Bradford prospective birth cohort.

The exposure of two or more pre-existing, long-term physical or mental health conditions will be defined from a list of health conditions predetermined by women and clinicians. The association of maternal multiple long-term conditions with (i) antenatal, (ii) peripartum, (iii) postnatal and long-term, and (iv) mental health outcomes, for both women and their children will be examined. Outcomes of interest will be guided by a core outcome set.

Comparisons will be made between pregnant women with and without multiple long-term conditions using logistic and Cox regression. Generalised estimating equation will account for the clustering effect of women who had more than one pregnancy episode. Where appropriate, multiple imputation with chained equation will be used for missing data. Federated analysis will be conducted for each dataset and results will be pooled using meta-analysis.

Ethics and dissemination:

For peer review only - http://bmjopen.bmj.com/site/about/guidelines.xhtml

Approval has been obtained from the respective data sources in each UK nation. Study findings will be submitted for publications in peer reviewed journals and presented at key conferences.

282 words

For peer review only

For peer review only - http://bmjopen.bmj.com/site/about/guidelines.xhtml

ARTICLE SUMMARY

Strengths and limitations of this study

- The study will utilise rich data sources from routine health records from all four UK nations and a birth cohort.
- Beyond examining maternal outcomes, linked mother baby data and the birth cohort data will allow for the exploration of children's outcomes.
- Key limitations include missing data, misclassification bias due to inaccurate clinical coding and residual confounding.

For peer review only

INTRODUCTION

One in five pregnant women have two or more long-term physical or mental health conditions prior to pregnancy in the United Kingdom (UK).[1] In the UK 2016-18 national maternal mortality report, 90% of women who died during or up to a year after pregnancy had multiple health or social problems.[2] Recent evidence has shown that maternal multiple long-term conditions are associated with adverse outcomes for women and their children, such as severe maternal morbidity and mortality, pre-eclampsia, emergency caesarean birth, preterm birth, and low birth weight.[3-5]

Information on consequences for women with multiple long-term conditions and their children is crucial for women and their health care professionals to make informed decisions on pregnancy care planning. However, there remains a lack of evidence to guide care pathways for pregnant women with multiple long-term conditions.[5 6]

Healthcare is free in the UK and over 98% of the population are registered at a general practice (akin to family practice in other countries).[7] General practices not only provide primary and community healthcare, but they also serve as the main point of contact for referrals to specialist clinical services and provide the majority of prescribing outside of a hospital setting.[7] In the UK, pregnant women are recommended to have their booking appointment before 10 weeks gestation.[8] This is the pregnant woman's first midwife or doctor appointment, where they undergo health and social care assessment of needs and risks for her pregnancy.[9] Over 97% of births occur in healthcare settings in England and Wales.[10] Therefore, routine health records in primary and secondary care in the UK offer a rich data source for observational studies of pregnant women and their children.

This observational study aims to compare outcomes for women with multiple long-term conditions to those without multiple long-term conditions. Outcomes studied will include

For peer review only - http://bmjopen.bmj.com/site/about/guidelines.xhtml

those for women and their children. Datasets from routine health records from all four UK nations (England, Wales, Scotland, and Northern Ireland) will be used. In addition, the Born in Bradford birth cohort from a deprived, ethnically diverse city in the UK, will also be used.[11]

The four research objectives are to examine the association between maternal pre-existing multiple long-term conditions with: (1) antenatal, (2) peripartum, (3) postnatal and long-term outcomes, and (4) mental health outcomes. The findings from each research objective will be published in a separate paper.

METHODS AND ANALYSIS

Study design

This is an observational study using data from routine healthcare records and a prospective birth cohort in the UK.

Study population and eligibility criteria

The study population will consist of women aged 15-49 years old at conception, with pregnancies beginning between 2000 and 2019 in the UK. Date of conception (pregnancy start date) will be defined as the first day of the last menstrual period or gestational day 0. To ensure sufficient quality data, eligible women must have health records that meet the standard data quality checks as defined by each data source and one year's worth of health records prior to index pregnancy.

For peer review only - http://bmjopen.bmj.com/site/about/guidelines.xhtml

Data sources

Five population-based data sources (four routine health record datasets and one prospective birth cohort) that will be used are described as follows.

(1) Clinical Practice Research Datalink (CPRD), England

CPRD contains anonymised, longitudinal medical records collected during provision of routine healthcare, from participating general practices in the UK; currently 5% of UK general practices contributes to the database.[12] It includes data on demographics, diagnoses, symptoms, signs, tests and prescriptions.[7] Linkage to area based deprivation index, Hospital Episodes Statistics and Office for National Statistics death registration data is available for patients whose general practices have consented to the CPRD linkage scheme.[7]

Within CPRD, the CPRD Pregnancy Register is an algorithm that takes information from maternity, antenatal and birth health records from primary care to detect pregnancy episodes and their outcomes.[13] The Mother Baby Linked data, similarly links women and child records using an algorithm,[14] will allow for studying the outcomes of children born to mothers with multiple long-term conditions.

CPRD has data for patients from all four UK nations, but analyses using CPRD data will only include English general practices to avoid duplication of patients with datasets from the devolved nations (Wales, Scotland and Northern Ireland).

(2) Secure Anonymised Information Linkage (SAIL), Wales

The SAIL databank is a whole population level database in Wales. It is a repository of anonymised health and socio-economic administrative data and provides linkage at an individual level.[15] It holds data for 4.8 million people and covers 80% of Welsh general practices.[15] Within SAIL, the National Community Child Health Dataset will be used to

For peer review only - http://bmjopen.bmj.com/site/about/guidelines.xhtml

detect births and linked to the Welsh Longitudinal General Practice dataset and the Welsh Demographic Service dataset for diagnoses, prescriptions and demographics data respectively.

(3) Scotland

A dataset will be created linking the Scottish Maternity Records (SMR02) to data from Hospital Admissions (SMR01), Mental Health Inpatients (SMR04), Accident and Emergency, and the Demography and Death registry. This will cover diagnoses and demographic data for all inpatient stays and day cases for residents in Scotland. The dataset will also be linked to the Prescribing Information System for data on all medications dispensed in the community. Pregnancies will be detected from maternity records or pregnancy-related hospital admissions.

(4) Northern Ireland Maternity System (NIMATS)

NIMATS holds demographic and clinical information on mothers and infants.[16] It captures data relating to the current complete maternity process, and the women's past medical and obstetric history.[16] It is a key source for data on birth numbers, interventions, maternal risk factors, birth weights, maternal smoking, body mass index and breastfeeding on discharge.[16] NIMATS covers all five Health and Social Care Trusts areas across Northern Ireland (11 hospitals providing maternity services in total).[16] Access to NIMATS is also available to midwives and clerical staff in various community clinics across NI to allow for booking appointments to be recorded.[16] Pregnancies will be determined from maternity records derived from data recorded at booking appointments (the first antenatal appointment) and hospital admission for childbirth.

For peer review only - http://bmjopen.bmj.com/site/about/guidelines.xhtml

(5) Born in Bradford

This birth cohort follows over 13,500 children born from around 12,500 mothers at the Bradford Royal Infirmary between March 2007 and June 2011.[17] Data is collected from pregnancy through childhood and into adult life.[17] The database consists of over 13,500 pregnancies with biological samples, sociodemographic data, offspring developmental, clinical and education data and is linked to health care records from maternity, primary care (mother and offspring) and hospital admissions.[17]

Exposure

The exposed group will consist of pregnant women with multiple long-term conditions. We shall define this as two or more long-term physical or mental health conditions that pre-existed before pregnancy. Pregnancy related complications will not be included as they will be studied as outcomes. Multiple long-term conditions will be defined from a list of health conditions previously described in our epidemiological work.[1] Sensitivity analysis will be performed defining maternal long-term conditions with a different list of health conditions by D'Arcy et al.[18]

Exposure will be ascertained by the presence of diagnostic or prescriptions codes, including Read (to identify exposures in primary care data), International Classification of Disease 10th version (ICD-10, secondary care) and Operating Procedures Codes (OPCS) Classification of Interventions and Procedures.

For peer review only - http://bmjopen.bmj.com/site/about/guidelines.xhtml

Comparator

Pregnant women with no multiple long-term conditions (i.e. no or single long-term conditions) will be the comparator group. Comparisons will be made with the following exposure group:

(i) pregnant women with multiple long-term conditions;

(ii) pregnant women with increasing counts of long-term health conditions;

(iii) pregnant women with different combinations of long-term health conditions.

(iv) pregnant women in different health condition clusters (identified from ongoing clustering analyses); and

In addition, we will also compare the outcomes for pregnant women who have mental health conditions as part of their multiple long-term conditions with pregnant women with multiple long-term conditions who do not have mental health conditions.

Outcomes

The outcomes will be grouped into the following four categories based on the research objectives: (1) antenatal, (2) peripartum, (3) postnatal and long-term outcomes, and (4) mental health outcomes. Examples of outcomes are provided as follows, based on existing core outcome sets for pregnancy and childbirth.[19 20] The definitive list of outcomes will be confirmed once the development work for a core outcome set for studies of pregnant women with multiple long-term conditions is completed.[21] Outcomes will be ascertained using Read, ICD-10 and OPCS codes.

For peer review only - http://bmjopen.bmj.com/site/about/guidelines.xhtml

(1) Antenatal

Antenatal outcomes occur from conception to before the onset of childbirth. Examples for women include miscarriage, gestational hypertension, pre-eclampsia, gestational diabetes, venous thromboembolism and placenta abruption. Examples for children include fetal growth restriction.

(2) Peripartum

Peripartum outcomes occur during and immediately after childbirth. This category will also include survival outcomes for women and children. Examples for women include mode of birth (spontaneous vaginal birth , birth with forceps/ ventouse, caesarean birth), postpartum haemorrhage, severe maternal morbidity and maternal death. Examples for children include preterm birth, small for gestational age, stillbirth, perinatal death and neonatal death.

(3) Postnatal and long-term

Postnatal outcomes occur in the 42 days after birth.[22] We will also include perinatal health care utilisation outcomes and long-term outcomes enduring beyond the peripartum and postpartum period. Examples for women include incontinence. Examples for children include congenital anomaly and neurodevelopmental disorders. Examples for health care utilisation include admission to intensive care.

(4) Mental health

Mental health outcomes cover the antenatal and postnatal period. Mental health outcomes will be considered up to 12 months after birth. This is to account for possible delay in women presenting to clinicians and reaching a formal diagnosis. Examples include postnatal depression, puerperal psychosis, post-traumatic stress disorder, self-harm and suicide attempts. Children's mental health and behavioural disorders will also be considered.

For peer review only - http://bmjopen.bmj.com/site/about/guidelines.xhtml

Covariates

Analyses will adjust for the following covariates in a hierarchical manner to explore potential mediating effects. Additional covariates may be added for individual outcomes based on the literature. Where data for antenatal exposures are available (e.g. from NIMATS and Born in Bradford's booking appointments), additional analyses may be conducted where appropriate.

(i) Maternal age

We shall explore whether the association between maternal age and the outcomes are linear. Where this is not the case and to aid clinical interpretability, we will categorise maternal age at conception into 5-yearly age bands.

(ii) Parity/gravidity

The variable used will depend on availability in study datasets. Where both variables are available, both will be reported with preference given to *parity (*the number of times a woman gave birth at gestation ≥24 weeks); and sensitivity analysis will be conducted using *gravidity (*the number of times a woman has been pregnant).

(iii) Ethnicity

Maternal ethnicity will be categorised based on the variables available in the study datasets: Asian, Black, Mixed, Other and White. Where numbers are too small and risk identifying individuals, such as in NIMATS, we may collapse the categories to White and Non-white.

(iv) Social deprivation

The patient level Index of Multiple Deprivation specific to each nation will be used and categorised into quintiles.

For peer review only - http://bmjopen.bmj.com/site/about/guidelines.xhtml

(v) Body mass index

We shall include the latest available pre-pregnancy body mass index (BMI) for the pregnant women. Where booking data is available before 16 weeks gestation, this will be used (e.g. in NIMATS). BMI will be considered a covariate instead of a health condition. The World Health Organisation's classification of obesity will be used to categorise BMI: <18.5 kg/m^2, 18.5 to 24.9 kg/m^2, 25.0 to 29.9 kg/m^2, 30.0 to 34.9 kg/m^2, 35.0 to 39.9 kg/m^2, and 40+ kg/m^2.[23] Categories may be combined where numbers are too small.

(vi) Smoking

We shall include the latest available pre-pregnancy smoking status for the pregnant women. Smoking status will be categorised as: non-smoker, ex-smoker, and smoker.

(vii) Year (pregnancy start date)

Data quality and clinical guidelines may vary by year. Its effect on outcomes will be accounted for by adjusting for year of conception in the analysis.

Statistical analysis

Baseline characteristics of the study population and outcomes will be described with summary statistics. Multivariable logistic regression will be performed to estimate the odds ratios for selected study outcomes. Cox regression will be performed for longer-term outcomes. The unit of analysis will be the pregnancy episode.

The covariates will be adjusted for in a hierarchical manner. This is because our prior epidemiological study observed that BMI and smoking may mediate the association between multiple long-term conditions with social deprivation.[1]

For peer review only

For peer review only - http://bmjopen.bmj.com/site/about/guidelines.xhtml

A federated analysis approach will be used, each dataset will be analysed separately within the approval of the data access. The effect sizes from the different datasets will then be included in a meta-analysis to produce a summary measure.

Where rare combinations of health conditions and outcomes may lead to identification of an individual or at the prespecified minimum count allowed by each data source, we will suppress the output.

Pregnant women with more than one pregnancy episode

An individual may have more than one pregnancy over the study period. The pregnancy episodes of the same woman will not be independent of each other. The severity of the exposure variable (pre-existing multiple long-term conditions) may increase in later pregnancy episodes as the pregnant women accumulates more long-term health conditions. If a woman had an adverse pregnancy outcome, she is more at risk of the same adverse outcome in subsequent pregnancy episodes. We shall account for this clustering effect of women with more than one pregnancy episode during the study period using the Generalised Estimating Equation in the regression analyses.

Multiple pregnancies

The main analysis will be limited to singleton pregnancies. Outcomes for pregnant women with multiple long-term conditions and multiple pregnancies (i.e. twins and higher order pregnancies) will be analysed as a separate cohort.

For peer review only - http://bmjopen.bmj.com/site/about/guidelines.xhtml

Missing data

Where exposure and outcome conditions are identified based on diagnostic codes, the absence of the code will be considered as an absence of the condition. The level and types of missingness of covariates will be reviewed and where appropriate will be addressed with representing missing data as a separate category or multiple imputation with chain equation (MICE). For variables required to compute an outcome, missing values will be imputed using MICE. Example of these variables include birthweight, gestational age and baby's sex to determine preterm birth and small for gestational age. For each outcome, the statistical analyses will be performed on the imputed datasets and the estimates will be pooled with Rubin's rule.

Sensitivity analysis

We shall conduct sensitivity analysis using (i) complete case analysis and (ii) varying definitions of maternal multiple long-term conditions exposure using D'Arcy et al's core exposure set.[18]

Patient and public involvement

The research question was informed by discussions with our patient and public involvement (PPI) advisory group and our PPI co-investigators NM and RP.

The selection of outcomes are guided by our ongoing work developing a core outcome set for studies of pregnant women with multiple long-term conditions, where patients are key stakeholders.[21]

For peer review only - http://bmjopen.bmj.com/site/about/guidelines.xhtml

Our PPI advisory group and PPI co-investigators will be involved in interpreting the study findings, producing lay summaries and infographics, and disseminating the study findings through their network.

Dissemination

Study findings will be submitted for publications in peer reviewed journals and presented at key conferences for health and social care professionals involved in the care of pregnant women with multiple long-term conditions and their children. We will also organise dissemination events to share our findings with the public, service users, clinicians and researchers.

DISCUSSION

MuM-PreDiCT is a consortium across all four nations of the UK studying multiple long-term conditions in pregnancy. As part of MuM-PreDiCT's program of work, we outlined the protocol for an observational study of maternal and children's outcome for pregnant women with multiple long-term conditions, using routine health records and a birth cohort in the UK.

Comparison with current literature

A recent systematic review found seven observational studies on the association of pre-pregnancy multiple long-term conditions with adverse maternal outcomes.[5] The review found that pre-pregnancy multiple long-term conditions were associated with severe maternal morbidity, hypertensive disorders of pregnancy, and acute health care use in the perinatal period.[5] Most studies were conducted in the United States.[5] Authors of the review

For peer review only - http://bmjopen.bmj.com/site/about/guidelines.xhtml

commented that many studies included conditions arising in pregnancy in defining multiple long-term conditions, making it difficult to examine the impact of chronic conditions on maternal health.[5]

This proposed study will be based in the UK and will use a broad range of long-term conditions selected by women and clinicians to define multiple long-term conditions. Pregnancy related conditions and complications will be treated as study outcomes and not included in the exposure's definition. We will also study outcomes across all stages of pregnancy and outcomes for both women and their children.

Strengths and limitations

This proposed study will utilise routine health records from all four nations of the UK (England, Scotland, Wales and Northern Ireland). The available data sources consist of anonymised patient records from primary and secondary care, community prescription data, and maternity care data from routine booking appointments (first antenatal appointment offered universally and as the gateway to access maternity care in the UK).

Rich data will also be available from a prospective birth cohort from Bradford, an ethnically diverse population in England. Beyond examining maternal outcomes, linked mother baby data and the birth cohort data will allow for the exploration of children's outcomes. The key strength of this proposed study therefore is the generalisability of study findings to the UK population.

As this is an observational study using anonymised routine health records, key limitations include missing data, misclassification bias due to inaccurate clinical coding and residual confounding.

For peer review only - http://bmjopen.bmj.com/site/about/guidelines.xhtml

Clinical implications

Current obstetric guidelines for pregnant women with medical conditions are focused on specific and single health conditions.[24] There are currently no guidelines for the management of pregnant women with multiple long-term conditions in the UK. As observed in the systematic review, there is currently a lack of evidence on the consequences of pregnancy for women with multiple long-term conditions.[5] Our PPI advisory group and preliminary findings from our core outcome set development work have highlighted how women valued having information to help them mentally prepare to face potential adverse pregnancy outcomes.

The output from this study will therefore provide valuable information for women to make informed decision with their clinicians about family planning and their preconception, pregnancy and postpartum care. It will also provide valuable information to guide the future design of care pathway for women with multiple long-term conditions.

Conclusion

This protocol outlines the study design of an observational study quantifying maternal and children's outcomes for pregnant women with multiple long-term conditions. The outputs from this study will add to the current body of literature and provide valuable information to help women and their clinicians with their preconception, pregnancy and postpartum care planning.

For peer review only - http://bmjopen.bmj.com/site/about/guidelines.xhtml

ABBREVIATIONS

BMI	Body mass index
CPRD	Clinical Practice Research Datalink
ICD-10	International Classification of Disease 10th version
MICE	Multiple imputation with chain equation
NICE	National Institute for Health and Care Excellence
NIMATS	Northern Ireland Maternity System
OPCS	Operating Procedures Codes
PPI	Patient and public involvement
SAIL	Secure Anonymised Information Linkage
UK	United Kingdom

For peer review only

For peer review only - http://bmjopen.bmj.com/site/about/guidelines.xhtml

Ethics approval

CPRD: CPRD has broad National Research Ethics Service Committee ethics approval for purely observational research using the primary care data and established data linkages. The study has been reviewed and approved by CPRD's Independent Scientific Advisory Committee (reference: 20_181R).

SAIL: In accordance with UK Health Research Authority guidance, ethical approval is not mandatory for studies using only anonymised data. The study has been approved by SAIL Information Governance Review Panel.

Scotland dataset: The study has been approved by the National Health Service Scotland Public Benefit and Privacy Panel for Health and Social Care (HSC-PBPP) and The University Teaching and Research Ethics Committee (UTREC) from the University of St Andrews.

NIMATS: The study has been approved by the Honest Broker Service Governance Board.

Born in Bradford: Ethics approval was granted by Bradford National Health Service Research Ethics Committee (ref 07/H1302/112) for the Born in Bradford cohort.

The proposed study is purely observational and will use anonymised research data. The study will not involve participant recruitment. Therefore, consent to participate is not required.

Consent for publication

This is not applicable as the manuscript is a study protocol. In the proposed study, we will use de-identified study data, therefore consent for publication will not be required.

For peer review only - http://bmjopen.bmj.com/site/about/guidelines.xhtml

Availability of data and materials

This is not applicable as the manuscript is a study protocol. In the proposed study, the data that support the findings are available from CPRD, SAIL, Scotland National Health Service Scotland Public Benefit and Privacy Panel for Health and Social Care, NIMATS and Born in Bradford, but restrictions apply to the availability of these data, which were used under license for the current study, and so are not publicly available. Data are however available from the authors upon reasonable request and with permission of CPRD, SAIL, Scotland National Health Service Scotland Public Benefit and Privacy Panel for Health and Social Care, NIMATS and Born in Bradford.

Competing interests

The authors declare that they have no competing interests.

Funding

This work is funded by the Strategic Priority Fund "Tackling multimorbidity at scale" programme (grant number MR/W014432/1) delivered by the Medical Research Council and the National Institute for Health Research (NIHR) in partnership with the Economic and Social Research Council and in collaboration with the Engineering and Physical Sciences Research Council. The views expressed are those of the author and not necessarily those of the funders, the NIHR or the UK Department of Health and Social Care. The funders had no role in study design, decision to publish, or preparation of the manuscript.

Author contributions

SIL – Conceptualisation, funding acquisition, methodology, writing (original draft preparation).

KN, MB, KAE, KMA, DOR - Conceptualisation, funding acquisition, methodology, supervision, writing (review and editing)

For peer review only

HH, GS, AS, NM, AAL, AFF, CNP, CY, CMC, JIK, PB, RP, RR, ST, SB, UA, ZV- Conceptualisation, funding acquisition, methodology, writing (review and editing)

LK, KP, MS, MM, NC, SPBHS - Conceptualisation, methodology, writing (review and editing)

All authors read and approved the manuscript.

Acknowledgements

Not applicable.

For peer review only

For peer review only - http://bmjopen.bmj.com/site/about/guidelines.xhtml

REFERENCES

1. Lee SI, Azcoaga-Lorenzo A, Agrawal U, et al. Epidemiology of pre-existing multimorbidity in pregnant women in the UK in 2018: a population-based cross-sectional study. *BMC pregnancy and childbirth* 2022;22(1):120. doi: 10.1186/s12884-022-04442-3
2. Knight M, Bunch K, Tuffnell D, et al. Saving Lives, Improving Mothers' Care - Lessons learned to inform maternity care from the UK and Ireland Confidential Enquiries into Maternal Deaths and Morbidity 2016-18. Oxford: National Perinatal Epidemiology Unit, University of Oxford, 2020.
3. Admon LK, Winkelman TNA, Heisler M, et al. Obstetric Outcomes and Delivery-Related Health Care Utilization and Costs Among Pregnant Women With Multiple Chronic Conditions. *Preventing Chronic Disease* 2018;15:E21.
4. D'Arcy R, Knight M, Mackillop L. A retrospective audit of the socio-demographic characteristics and pregnancy outcomes for all women with multiple medical problems giving birth at a tertiary hospital in the UK in 2016. *BJOG: An International Journal of Obstetrics and Gynaecology* 2019;126:128.
5. Brown HK, McKnight A, Aker A. Association between pre-pregnancy multimorbidity and adverse maternal outcomes: A systematic review. *Journal of multimorbidity and comorbidity* 2022;12:26335565221096584. doi: 10.1177/26335565221096584 [published Online First: 2022/05/20]
6. Beeson JG, Homer CSE, Morgan C, et al. Multiple morbidities in pregnancy: Time for research, innovation, and action. *PLoS medicine* 2018;15(9):e1002665. doi: 10.1371/journal.pmed.1002665 [published Online First: 2018/09/27]
7. Herrett E, Gallagher AM, Bhaskaran K, et al. Data Resource Profile: Clinical Practice Research Datalink (CPRD). *International Journal of Epidemiology* 2015;44(3):827-36. doi: 10.1093/ije/dyv098
8. National Institute for Health and Care Excellence. Clinical Knowledge Summaries: Antenatal Care - Uncomplicated Pregnancy. 2021 [Available from: https://cks.nice.org.uk/topics/antenatal-care-uncomplicated-pregnancy/management/antenatal-care-uncomplicated-pregnancy/#booking-appointment accessed 5th July 2022.
9. Hatherall B, Morris J, Jamal F, et al. Timing of the initiation of antenatal care: An exploratory qualitative study of women and service providers in East London. *Midwifery* 2016;36:1-7. doi: 10.1016/j.midw.2016.02.017 [published Online First: 2016/04/24]
10. Office for National Statistics. Birth characteristics in England and Wales: 2020. 2022 [Available from: https://www.ons.gov.uk/peoplepopulationandcommunity/birthsdeathsandmarriages/livebirths/bulletins/birthcharacteristicsinenglandandwales/2020#place-of-birth accessed 12th July 2022.
11. Raynor P. Born in Bradford, a cohort study of babies born in Bradford, and their parents: protocol for the recruitment phase. *BMC public health* 2008;8:327. doi: 10.1186/1471-2458-8-327 [published Online First: 2008/09/25]
12. CPRD. Release notes: CPRD GOLD May 2022. 2022 [Available from: https://cprd.com/sites/default/files/2022-05/2022-05%20CPRD%20GOLD%20Release%20Notes.pdf accessed 14th July 2022.
13. Minassian C, Williams R, Meeraus WH, et al. Methods to generate and validate a Pregnancy Register in the UK Clinical Practice Research Datalink primary care database. *Pharmacoepidemiology and drug safety* 2019;28(7):923-33. doi: 10.1002/pds.4811 [published Online First: 2019/06/15]
14. Clinical Practice Research Datalink. CPRD Mother Baby Link Documentation 2017 [Available from: https://cprd.com/sites/default/files/2022-02/MotherBabyLink_Documentation_v1.2.pdf accessed 8th August 2022.
15. SAIL Databank. 10 years of spearheading data privacy and research utility [cited 2021 16th February]. Available from: https://saildatabank.com/wp-content/uploads/SAIL_10_year_anniversary_brochure.pdf.

For peer review only - http://bmjopen.bmj.com/site/about/guidelines.xhtml

16. Health Data Research Innovation Gateway. Northern Ireland Regional Maternity system (NIMATS) 2021 [cited 2021 7 December]. Available from: https://web.www.healthdatagateway.org/dataset/89d76819-32cb-48ad-9337-880f324a6ec0.
17. Born in Bradford. About Us 2021 [cited 2021 7 December]. Available from: https://borninbradford.nhs.uk/about-us/.
18. D'Arcy R, Knight M. List of health conditions to define multiple long-term conditions in pregnancy. [Available from: https://drive.google.com/file/d/1Q3LTzcXGnEFcS6eMAA0pntEUUUa-iTtp/view accessed 14th July 2022.
19. Devane D, Begley CM, Clarke M, et al. Evaluating maternity care: a core set of outcome measures. *Birth (Berkeley, Calif)* 2007;34(2):164-72. doi: 10.1111/j.1523-536X.2006.00145.x [published Online First: 2007/06/05]
20. Nijagal MA, Wissig S, Stowell C, et al. Standardized outcome measures for pregnancy and childbirth, an ICHOM proposal. *BMC Health Services Research* 2018;18(1):953. doi: 10.1186/s12913-018-3732-3
21. Lee SI, Eastwood K-A, Moss N, et al. Protocol for the development of a core outcome set for studies of pregnant women with pre-existing multimorbidity. *BMJ Open* 2021;11(10):e044919. doi: 10.1136/bmjopen-2020-044919
22. World Health Organization. WHO Technical Consultation on Postpartum and Postnatal Care,. 2010. https://www.ncbi.nlm.nih.gov/books/NBK310595/ (accessed 6th July 2022).
23. World Health Organization. Obesity: preventing and managing the global epidemic. Report of a WHO consultation. *World Health Organization technical report series* 2000;894:i-xii, 1-253. [published Online First: 2001/03/10]
24. National Institute for Health and Care Excellence. NICE guideline [NG121]: Intrapartum care for women with existing medical conditions or obstetric complications and their babies 2019 [Available from: https://www.nice.org.uk/guidance/ng121 accessed 8th July 2022.

For peer review only

For peer review only - http://bmjopen.bmj.com/site/about/guidelines.xhtml

BMJ Open

Maternal and child outcomes for pregnant women with pre-existing multiple long-term conditions: protocol for an observational study in the United Kingdom

Journal:	*BMJ Open*
Manuscript ID	bmjopen-2022-068718.R1
Article Type:	Protocol
Date Submitted by the Author:	09-Jan-2023

Complete List of Authors:	Lee, Siang Ing; University of Birmingham Institute of Applied Health Research<br>Hope, Holly; The University of Manchester, Centre for Women's Mental Health, Faculty of Biology Medicine & Health<br>OReilly, Dermot; Queen's University Belfast, Centre for Public Health<br>Kent, Lisa; Queen's University Belfast, Centre for Public Health<br>Santorelli, Gillian; Bradford Institute for Health Research<br>Subramanian, Anuradhaa; University of Birmingham Institute of Applied Health Research<br>Moss, Ngawai; Patient and public representative<br>Azcoaga-Lorenzo, Amaya; University of St Andrews School of Medicine, Division of Population and Behavioural Sciences; Hospital Rey Juan Carlos, Instituto de Investigación Sanitaria Fundación Jimenez Diaz<br>Fagbamigbe, Adeniyi; University of St Andrews School of Medicine, Division of Population and Behavioural Sciences; University of Ibadan College of Medicine, Department of Epidemiology and Medical Statistics<br>Nelson-Piercy, Catherine; Guy's and St Thomas' NHS Foundation Trust<br>Yau, Christopher; University of Oxford, Nuffield Department of Women's and Reproductive Health; Health Data Research UK<br>McCowan, Colin ; University of St Andrews School of Medicine, Division of Population and Behavioural Sciences<br>Kennedy, Jonathan; Swansea University, Data Science, Medical School<br>Phillips, Katherine; University of Birmingham Institute of Applied Health Research<br>Singh, Megha; University of Birmingham Institute of Applied Health Research<br>Mhereeg, Mohamed; Swansea University, Data Science, Medical School<br>Cockburn, Neil; University of Birmingham Institute of Applied Health Research<br>Brocklehurst, Peter; University of Birmingham Institute of Applied Health Research<br>Plachcinski, Rachel; Patient and public representative<br>Riley, Richard; University of Birmingham Institute of Applied Health Research<br>Thangaratinam, Shakila; University of Birmingham Institute of Metabolism and Systems Research, WHO Collaborating Centre for Global Women's Health; Birmingham Women's and Children's NHS Foundation Trust, Department of Obstetrics and Gynaecology<br>Brophy, Sinead; Swansea University, Data Science, Medical School

	Hemali Sudasinghe, Sudasing Pathirannehelage Buddhika; University of Birmingham Institute of Applied Health Research<br>Agrawal, Utkarsh; University of St Andrews School of Medicine, Division of Population and Behavioural Sciences<br>Vowles, Zoe; Guy's and St Thomas' NHS Foundation Trust<br>Abel, Kathryn ; The University of Manchester, Centre for Women's Mental Health, Faculty of Biology Medicine & Health; Greater Manchester Mental Health NHS Foundation Trust<br>Nirantharakumar, Krishnarajah; University of Birmingham Institute of Applied Health Research<br>Black, Mairead; University of Aberdeen School of Medicine Medical Sciences and Nutrition, Aberdeen Centre for Women’s Health Research<br>Eastwood, Kelly-Ann; Queen's University Belfast, Centre for Public Health; University Hospitals Bristol and Weston NHS Foundation Trust<br>-, on behalf of MuM-PreDiCT; MuM-PreDiCT

**Primary Subject Heading**:	Obstetrics and gynaecology
Secondary Subject Heading:	Epidemiology
Keywords:	Maternal medicine < OBSTETRICS, OBSTETRICS, EPIDEMIOLOGY

SCHOLARONE™
Manuscripts

Title: Maternal and child outcomes for pregnant women with pre-existing multiple long-term conditions: protocol for an observational study in the United Kingdom

Authors:

Siang Ing Lee,[1] Holly Hope,[2] Dermot O'Reilly,[3] Lisa Kent,[3] Gillian Santorelli,[4] Anuradhaa Subramanian,[1] Ngawai Moss,[5] Amaya Azcoaga-Lorenzo,[6, 7] Adeniyi Francis Fagbamigbe,[6, 8] Catherine Nelson-Piercy,[9] Christopher Yau,[10,11] Colin McCowan,[6] Jonathan I Kennedy,[12] Katherine Phillips,[1] Megha Singh,[1] Mohamed Mhereeg,[12] Neil Cockburn,[1] Peter Brocklehurst,[1] Rachel Plachcinski,[5] Richard Riley,[1] Shakila Thangaratinam,[13, 14] Sinead Brophy,[12] Sudasing Pathirannehelage Buddhika Hemali Sudasinghe,[1] Utkarsh Agrawal,[6] Zoe Vowles,[9] Kathryn M Abel,[2, 15] * Krishnarajah Nirantharakumar,[1]* Mairead Black,[16]* Kelly-Ann Eastwood,[3, 17]* on behalf of the MuM-PreDiCT Group.

*Joint senior authors

Corresponding author: Krishnarajah Nirantharakumar, K.Nirantharan@bham.ac.uk

Affiliation:

1. Institute of Applied Health Research, University of Birmingham, Birmingham, UK
2. Centre for Women's Mental Health, Faculty of Biology Medicine & Health, The University of Manchester, UK
3. Centre for Public Health, Queen's University of Belfast, UK
4. Bradford Institute for Health Research, UK
5. Patient and public representative, UK
6. Division of Population and Behavioural Sciences, School of Medicine, University of St Andrews, UK
7. Hospital Rey Juan Carlos. Instituto de Investigación Sanitaria Fundación Jimenez Diaz. Madrid. Spain
8. Department of Epidemiology and Medical Statistics, College of Medicine, University of Ibadan, Nigeria
9. Guy's and St. Thomas' NHS Foundation Trust, UK
10. Nuffield Department of Women's and Reproductive Health, University of Oxford, UK
11. Health Data Research UK
12. Data Science, Medical School, Swansea University, UK
13. WHO Collaborating Centre for Global Women's Health, Institute of Metabolism and Systems Research, University of Birmingham, UK
14. Department of Obstetrics and Gynaecology, Birmingham Women's and Children's NHS Foundation Trust, UK

For peer review only - http://bmjopen.bmj.com/site/about/guidelines.xhtml

15. Greater Manchester Mental Health NHS Foundation Trust, Manchester, UK
16. Aberdeen Centre for Women's Health Research, School of Medicine, Medical Science and Nutrition, University of Aberdeen, UK
17. St Michael's Hospital, University Hospitals Bristol NHS Foundation Trust, UK

Authors' ORCID:

Siang Ing Lee 0000-0002-2332-5452, Holly Hope 0000-0002-4834-6719, Dermot O'Reilly 0000-0002-9181-0652, Lisa Kent 0000-0002-8882-0526, Gillian Santorelli 0000-0003-0427-1783, Anuradhaa Subramanian 0000-0001-8875-7363, Ngawai Moss 0000-0001-9369-5072, Amaya Azcoaga-Lorenzo 0000-0003-3307-878X, Adeniyi Francis Fagbamigbe 0000-0001-9184-8258, Catherine Nelson-Piercy 0000-0001-9311-1196, Christopher Yau 0000-0001-7615-8523, Colin McCowan 0000-0002-9466-833X, Jonathan I Kennedy 0000-0002-1122-6502, Katherine Phillips 0000-0003-0674-605X, Megha Singh 0000-0003-3680-7124, Mohamed Mhereeg 0000-0003-1241-9549, Neil Cockburn 0000-0001-9284-6991, Peter Brocklehurst 0000-0002-9950-6751, Rachel Plachcinski 0000-0001-9908-0773, Richard Riley 0000-0001-8699-0735, Shakila Thangaratinam 0000-0002-4254-460X, Sinead Brophy 0000-0001-7417-2858, Utkarsh Agrawal 0000-0001-5181-6120, Zoe Vowles 0000-0001-6989-2180, Kathryn M Abel 0000-0003-3538-8896, Krishnarajah Nirantharakumar 0000-0002-6816-1279, Mairead Black 0000-0002-6841-8601, Kelly-Ann Eastwood 0000-0003-3689-0490.

Main text word count: 3965

Keywords: multimorbidity, multiple chronic conditions, multiple long-term conditions, pregnancy, maternity, obstetric, outcome, children, offspring

review only

ABSTRACT

Introduction: One in five pregnant women have multiple pre-existing long-term conditions in the United Kingdom (UK). Studies have shown that maternal multiple long-term conditions are associated with adverse outcomes. This observational study aims to compare maternal and child outcomes for pregnant women with multiple long-term conditions to those without multiple long-term conditions (0 or 1 long-term conditions).

Methods and analysis: Pregnant women aged 15 to 49 years old with a conception date between 2000 and 2019 in the UK will be included with follow up till 2019. The data source will be routine health records from all four UK nations (Clinical Practice Research Datalink [CPRD, England], Secure Anonymised Information Linkage [SAIL, Wales], Scotland routine health records and Northern Ireland Maternity System [NIMATS]); and the Born in Bradford birth cohort. The exposure of two or more pre-existing, long-term physical or mental health conditions will be defined from a list of health conditions predetermined by women and clinicians. The association of maternal multiple long-term conditions with (i) antenatal, (ii) peripartum, (iii) postnatal and long-term, and (iv) mental health outcomes, for both women and their children will be examined. Outcomes of interest will be guided by a core outcome set. Comparisons will be made between pregnant women with and without multiple long-term conditions using modified Poisson and Cox regression. Generalised estimating equation will account for the clustering effect of women who had more than one pregnancy episode. Where appropriate, multiple imputation with chained equation will be used for missing data. Federated analysis will be conducted for each dataset and results will be pooled using random-effects meta-analyses.

Ethics and dissemination:

For peer review only - http://bmjopen.bmj.com/site/about/guidelines.xhtml

Approval has been obtained from the respective data sources in each UK nation. Study findings will be submitted for publications in peer reviewed journals and presented at key conferences.

295 words

For peer review only

For peer review only - http://bmjopen.bmj.com/site/about/guidelines.xhtml

ARTICLE SUMMARY

Strengths and limitations of this study

- The study will utilise rich data sources from routine health records from all four UK nations and a birth cohort.
- Beyond examining maternal outcomes, linked mother baby data and the birth cohort data will allow for the exploration of children's outcomes.
- Key limitations include missing data, misclassification bias due to inaccurate clinical coding and residual confounding.

For peer review only

For peer review only - http://bmjopen.bmj.com/site/about/guidelines.xhtml

INTRODUCTION

Maternal single long-term conditions such as cardiac conditions, chronic kidney disease and epilepsy are associated with adverse pregnancy outcomes.[1-4] This is likely to be compounded when the pregnant woman has two or more long-term physical or mental health conditions (multimorbidity). Some conditions may need different treatments from different health care teams, thereby increasing the treatment burden and complexity of care.[5] Recent evidence has shown that maternal multiple long-term conditions are associated with adverse outcomes for women and their children, such as severe maternal morbidity and mortality, pre-eclampsia, emergency caesarean birth, preterm birth, and low birth weight.[6-8] In the UK 2016-18 national maternal mortality report, 90% of women who died during or up to a year after pregnancy had multiple health or social problems.[9]

Currently one in five pregnant women have multiple long-term conditions prior to pregnancy in the United Kingdom (UK).[10] The number of pregnant women with pre-existing multiple long-term conditions is likely to increase as women are getting pregnant later in life and with higher body weight.[11-14] As this becomes an increasingly important issue, information on pregnancy, maternal and child outcomes is crucial for women and their health care professionals to make informed decisions on preconception and pregnancy care planning. However, there remains a lack of evidence to guide care pathways for pregnant women with multiple long-term conditions.[8 15]

Healthcare is free in the UK and over 98% of the population are registered at a general practice (akin to family practice in other countries).[16] General practices not only provide primary and community healthcare, but they also serve as the main point of contact for referrals to specialist clinical services and provide the majority of prescribing outside of a hospital setting.[16] In the UK, pregnant women are recommended to have their booking

For peer review only - http://bmjopen.bmj.com/site/about/guidelines.xhtml

appointment before 10 weeks gestation.[17] This is the pregnant woman's first midwife or doctor appointment, where they undergo health and social care assessment of needs and risks for her pregnancy.[18] Over 97% of births occur in healthcare settings in England and Wales.[19] Therefore, routine health records in primary and secondary care in the UK offer a rich data source for observational studies of pregnant women and their children.

This observational study aims to compare outcomes for women with multiple long-term conditions to those without multiple long-term conditions. Outcomes studied will include those for women and their children. Datasets from routine health records from all four UK nations (England, Wales, Scotland, and Northern Ireland) will be used. In addition, the Born in Bradford birth cohort from a deprived, ethnically diverse city in the UK, will also be used.[20]

The four research objectives are to examine the association between maternal pre-existing multiple long-term conditions with: (1) antenatal, (2) peripartum, (3) postnatal and long-term outcomes, and (4) mental health outcomes. The findings from each research objective will be published in a separate paper.

For peer review only

For peer review only - http://bmjopen.bmj.com/site/about/guidelines.xhtml

METHODS AND ANALYSIS

Study design

This is a cohort observational study using data from routine healthcare records and a birth cohort in the UK.

Study population and eligibility criteria

The study population will consist of women aged 15-49 years old at conception, with pregnancies beginning between 1st January 2000 and 31st December 2019 in the UK. Date of conception (pregnancy start date) will be defined as the first day of the last menstrual period or gestational day 0. To ensure sufficient quality data, eligible women must have health records that meet the standard data quality checks as defined by each data source and one year's worth of health records prior to index pregnancy.

Data sources

Table 1 presents the five data sources that will be used. Each UK devolved nation is represented by a population based routine health record dataset, with good national coverage for Wales, Scotland and Northern Ireland and a representative sample for England.[16] The exposure status will be determined from primary care records for Clinical Practice Research Datalink (CPRD) and Secure Anonymised Information Linkage (SAIL), with CPRD GOLD representing 5% of UK general practices,[21] and SAIL covering 80% of Welsh general practices.[22] For Scotland's linked routine records and Northern Ireland Maternity System (NIMATS), the exposure status will be determined from hospital and prescribing records.

For peer review only - http://bmjopen.bmj.com/site/about/guidelines.xhtml

CPRD and SAIL's primary care data offer the opportunity to study outcomes that may not be captured in secondary care. For instance, vomiting in pregnancy, miscarriage and neurodevelopmental conditions in children. The Scottish dataset provides detailed information on the different types of hospital attendances, including psychiatric admissions and accident and emergency attendances. NIMATS's unique first antenatal visit dataset is a good source of pre-pregnancy clinical data not available in other datasets.

As routine health records were not collected for research purposes, it is prone to missing data. Therefore, we have also included Born in Bradford, a regional birth cohort (2007-2011) where data were collected systematically and longitudinally from pregnancy, childhood through to adult life.

For peer review only

For peer review only - http://bmjopen.bmj.com/site/about/guidelines.xhtml

173 **Table 1: Summary of data sources**

Name of data source	Country	Population: pregnant women	Exposure: maternal multiple long-term conditions status	Outcomes: pregnant women	Outcomes: children
Clinical Practice Research Datalink (CPRD)[16]	England	Pregnancy register (primary care)	Primary care routine health records	Primary care records, hospital admissions, death registration	Mother-baby linked data: primary care records, hospital admissions, death registration
Secure Anonymised Information Linkage (SAIL)[22]	Wales	Births from National Community Child Health Dataset	Primary care routine health records	Primary care records, hospital admissions, death registration	Mother-baby linked data: primary care records, hospital admissions, death registration
Scotland routine health records	Scotland	Scottish Maternity Records, pregnancy-related hospital admissions	Hospital admissions, psychiatric admissions, accident and emergency attendances, prescriptions	Hospital admissions, psychiatric admissions, accident and emergency attendances, death registration	Mother-baby linked data: hospital admissions, psychiatric admissions, accident and emergency attendances, death registration
Northern Ireland Maternity System (NIMATS)[23]	Northern Ireland	Maternity booking (first antenatal) appointment records, birth related hospital admissions	Maternity booking (first antenatal) appointment records, birth related hospital admissions, prescriptions	Hospital admissions	Mother-baby linked data: hospital admissions
Born in Bradford[24]	Bradford, England	Birth cohort of over 13,500 children born from around 12,500 mothers at the Bradford Royal Infirmary between March 2007 and June 2011	Primary care routine health records	Data from birth cohort: clinical data<br>Data from linked health records: maternity, primary care, hospital admissions	Data from birth cohort: offspring developmental, clinical and education data<br>Data from linked health records: primary care, hospital admissions

For peer review only - http://bmjopen.bmj.com/site/about/guidelines.xhtml

Exposure

The exposed group will consist of pregnant women with multiple long-term conditions. Measurements of multiple long-term conditions are variable in existing literature.[25 26] Currently only Bateman et al's Maternal Comorbidity Index has been developed specifically for obstetric research.[27 28] It consists of 20 health conditions and included conditions arising in pregnancy such as gestational hypertension, pre-eclampsia and placenta praevia.[28] This limits the ability to study the impact of pre-existing long-term conditions on maternal and child health and the implication for long-term condition management preconception.[8]

In this study, we shall define multiple long-term conditions as two or more long-term physical or mental health conditions that pre-existed before pregnancy. Pregnancy related complications will not be included as they will be studied as outcomes. Multiple long-term conditions will be defined from a list of 79 health conditions previously described in our epidemiological work (Table 2) and will be measured with simple count.[10] This list was compiled from existing multimorbidity literature[9 26 29] and a workshop with our multidisciplinary research advisory group, including patient representatives and clinicians.[10] Selection of health conditions were based on: (i) prevalence; (ii) potential to impact on pregnancy outcomes; (iii) considered important by women; and (iv) recorded in the study datasets.[10] The phenome definitions for these health conditions have previously been described in our epidemiological work.[10] For health conditions that are transient and episodic in nature (e.g. asthma, eczema, depression and anxiety), we will only include the condition if it is active, which we have defined as requiring a doctors' consultation or medical prescription in the 12 months preceding pregnancy.[10] Sensitivity analysis will be performed defining maternal multiple long-term conditions with a different list of health conditions by D'Arcy et al.[30]

For peer review only - http://bmjopen.bmj.com/site/about/guidelines.xhtml

Exposure will be ascertained by the presence of diagnostic or prescriptions codes, including Read (to identify exposures in primary care data) and International Classification of Disease 10th version (ICD-10, secondary care).

Table 2: List of 79 health conditions defining multiple long-term conditions in pregnancy

Cancers

1. All cancers
   - Solid cancers
   - Haematological cancers
   - Metastatic cancers
   - Exclude basal cell carcinoma

Cardiovascular disease

2. Hypertension
3. Ischemic heart disease & myocardial infarction
4. Heart failure
5. Stroke
   - Transient ischemic attack
   - Ischemic stroke
   - Haemorrhagic stroke
   - Unspecified stroke
6. Atrial fibrillation
7. Congenital heart disease
8. Valvular heart disease (mitral, aortic, mixed)
9. Cardiomyopathy

Dermatology

10. Eczema
11. Psoriasis
12. Autoimmune skin disease
    - Vitiligo
    - Alopecia areata
13. Other dermatological conditions
    - Seborrheic dermatitis
    - Rosacea
    - Hidradenitis suppurativa
    - Lichen planus

Ear, Nose, Throat

14. Profound deafness
15. Allergic rhinitis & allergic conjunctivitis

Eye

16. Inflammatory eye disease
    - Scleritis & episcleritis
    - Anterior uveitis
    - Posterior uveitis
17. Cataract
18. Diabetic eye disease
19. Severe blindness
20. Retinal detachment

Neurodevelopmental conditions

43. Neurodevelopmental conditions
    - Learning disability
    - Attention deficit hyperactivity disorder
    - Autistic spectrum disorder

Rheumatology

44. Systemic lupus erythematosus
45. Spondylarthritis
    - Psoriatic arthritis
    - Ankylosing spondylitis
46. Inflammatory arthritis
    - Rheumatoid arthritis
    - Sjogern's syndrome
    - Raynaud's syndrome
    - Systemic sclerosis
    - Primary systemic vasculitis
47. Ehler's Danlos Syndrome (EDS) Type 3 (Hypermobile EDS)

Orthopaedic

48. Scoliosis
49. Vertebral disorder
    - Intervertebral disc disorder
    - Spondylosis
    - Spondylolisthesis
    - Collapsed vertebrae
    - Spinal stenosis
50. Chronic back pain
51. Osteoporosis
52. Osteoarthritis

Neurology

53. Migraine
54. Other chronic headache (including cluster headache, tension headache)
55. Epilepsy
56. Multiple sclerosis
57. Spina bifida
58. Idiopathic intracranial hypertension
59. Peripheral neuropathy
60. Other neurological conditions / musculoskeletal disorders
    - chronic fatigue syndrome / myalgic encephalomyelitis
    - fibromyalgia

For peer review only - http://bmjopen.bmj.com/site/about/guidelines.xhtml

**Gastroenterology**<br>21. Irritable bowel syndrome<br>22. Inflammatory bowel disease<br>○ Ulcerative colitis<br>○ Crohn's disease<br>23. Coeliac disease<br>24. Chronic liver disease<br>○ Chronic hepatitis B & C<br>○ Alcoholic liver disease<br>○ Autoimmune liver disease<br>○ Cirrhosis<br>○ Non-alcoholic fatty liver disease<br>25. Peptic ulcer<br>26. Gall stones<br><br>**Gynaecology**<br>27. Polycystic ovarian syndrome<br>28. Endometriosis<br>29. Fibroids<br>30. Infertility<br><br>**Haematology**<br>31. History of venous thromboembolism<br>○ Deep vein thrombosis<br>○ Pulmonary embolism<br>32. Primary thrombocytopenia<br>33. Haemophilia<br>34. Sickle cell anaemia<br>35. Pernicious anaemia<br><br>**Mental health**<br>36. Depression<br>37. Anxiety<br>○ Panic disorder<br>○ Phobia disorder<br>○ Post-traumatic stress disorder<br>38. Severe mental illness<br>○ Bipolar affective disorder<br>○ Schizophrenia<br>○ Psychosis<br>39. Eating disorder<br>40. History of alcohol use disorder (misuse / dependence)<br>41. History of substance misuse<br>42. Others<br>○ Obsessive compulsive disorder<br>○ Self-harm<br>○ Personality disorder<br>○ Dissociative disorder	○ chronic pain syndrome (includes chronic regional pain syndrome, myofascial pain syndrome)<br><br>**Respiratory**<br>61. Asthma<br>62. Chronic obstructive pulmonary disease<br>63. Obstructive sleep apnoea<br>64. Pulmonary fibrosis, interstitial lung disease<br>65. Pulmonary hypertension<br>66. Bronchiectasis<br>67. Cystic fibrosis<br>68. Sarcoidosis<br><br>**Renal**<br>69. Chronic kidney disease<br>70. Urinary tract stones<br><br>**Endocrine**<br>71. Diabetes mellitus<br>72. Thyroid disorder<br>73. Pituitary disorder<br>74. Adrenal benign tumour<br>75. Hyperparathyroidism<br><br>**Other**<br>76. Human immunodeficiency viral infection / Acquired immune deficiency syndrome<br>77. Turner's syndrome<br>78. Marfan's syndrome<br>**79.** Solid organ transplant

Comparator

Multiple long-term conditions versus no multiple long-term conditions

Comparisons will be made with the following exposure group:

For peer review only - http://bmjopen.bmj.com/site/about/guidelines.xhtml

(i) pregnant women with multiple long-term conditions;

(ii) pregnant women with increasing counts of long-term health conditions;

(iii) pregnant women with different combinations of long-term health conditions; and

(iv) pregnant women in different health condition clusters (identified from ongoing clustering analyses).

The selection of which combinations and clusters of long-term conditions to study will be based on how common they are and their clinical relevance, following consultation with patient representatives and clinicians in our research team. Pregnant women with no multiple long-term conditions (i.e. no or single long-term conditions) will be the common comparator group.

Multiple long-term conditions with and without mental illness

In addition, we will also compare the outcomes for pregnant women who have mental health conditions as part of their multiple long-term conditions against pregnant women with multiple long-term conditions who do not have mental health conditions.

Outcomes

The outcomes will be grouped into the following four categories based on the research objectives: (1) antenatal, (2) peripartum, (3) postnatal and long-term outcomes, and (4) mental health outcomes. Examples of outcomes are provided as follows, based on existing core outcome sets for pregnancy and childbirth.[31 32] The definitive list of outcomes will be confirmed once the development work for a core outcome set for studies of pregnant women with multiple long-term conditions is completed.[33] Outcomes will be ascertained from the study datasets (1st January 2000 to 31st December 2019) using clinical codes, such as Read,

For peer review only - http://bmjopen.bmj.com/site/about/guidelines.xhtml

ICD-10 and Operating Procedures Codes (OPCS) Classification of Interventions and Procedures.

(1) Antenatal

Antenatal outcomes occur from conception to before the onset of childbirth. Examples for women include miscarriage, gestational hypertension, pre-eclampsia, gestational diabetes, venous thromboembolism, placenta abruption and antenatal hospital admissions. Examples for children include fetal growth restriction.

(2) Peripartum

Peripartum outcomes occur during and immediately after childbirth. This category will also include survival outcomes for women and children. Examples for women include mode of birth (spontaneous vaginal birth , birth with forceps/ ventouse, caesarean birth), postpartum haemorrhage, severe maternal morbidity, admission to intensive care and maternal death. Examples for children include preterm birth, small for gestational age, admission to neonatal unit, stillbirth, perinatal death and neonatal death.

(3) Postnatal and long-term

Postnatal outcomes occur in the 42 days after birth,[34] while long-term outcomes are beyond the peripartum and postpartum period. For women this would include functional outcomes such as incontinence. For children, we will use mother baby linked primary and secondary care data to study postnatal and long-term outcomes such as congenital anomalies, neurodevelopmental conditions (e.g. autism, attention deficit hyperactive disorder and learning difficulty), cerebral palsy, and chronic lung disease. The length of follow up will depend on the availability of data in the routine health records. For example, CPRD has a median follow up of 5 years.[16] We will also examine postpartum readmission for mother and child.

For peer review only - http://bmjopen.bmj.com/site/about/guidelines.xhtml

(4) Mental health

Mental health outcomes cover the antenatal and postnatal period and will be considered up to 12 months after birth. This is to account for possible delay in women presenting to clinicians and reaching a formal diagnosis. We will consider both: (i) incident and (ii) recurrent mental health outcomes, where incident means a woman enters the analysis with no prior record of the specific mental health outcome. A perinatal mental health event is indicated by a primary care visit or hospital admission and includes mental health outcomes of concern in the antenatal and postnatal period (e.g.depression, psychosis, post-traumatic stress disorder, self-harm and suicide attempts). Comparing the mental health event rates of pregnant women who have and have not got mental health conditions as part of their multiple long-term conditions will allow us to delineate the contribution of mental and physical morbidity to perinatal mental health outcomes. Children's mental ill health will also be considered (e.g. depression and anxiety).

Covariates

Analyses will adjust for the following covariates. Additional covariates may be added for individual outcomes based on the literature. For example, in analyses of mental health outcomes there will be additional covariates. For the mother, we will include history of any mental illness, for the child we will include maternal history of any mental and/ or neurodevelopmental conditions.

Where data for antenatal exposures are available (e.g. from NIMATS and Born in Bradford's booking appointments), additional analyses may be conducted where appropriate.

(i) Maternal age

For peer review only - http://bmjopen.bmj.com/site/about/guidelines.xhtml

We shall explore whether the association between maternal age and the outcomes are linear. Where this is not the case and to aid clinical interpretability, we will categorise maternal age at conception into 5-yearly age bands.

(ii) Parity/gravidity

The variable used will depend on availability in study datasets. Where both variables are available, both will be reported with preference given to *parity (*the number of times a woman gave birth at gestation ≥24 weeks); and sensitivity analysis will be conducted using *gravidity* (the number of times a woman has been pregnant).

(iii) Ethnicity

Maternal ethnicity will be categorised based on the variables available and to allow for harmonisation across the datasets: Asian, Black, Mixed, Other and White. Where data permits, we may use more granular categories of ethnicity. Where numbers are too small and risk identifying individuals, such as in NIMATS, we may collapse the categories to White and Non-white.

(iv) Social deprivation

The patient level Index of Multiple Deprivation specific to each nation will be used and categorised into quintiles.

(v) Body mass index

We shall include the latest available pre-pregnancy body mass index for the pregnant women. Where booking data is available before 16 weeks gestation, this will be used (e.g. in NIMATS). Body mass index will be considered a covariate instead of a health condition. The World Health Organisation's classification of obesity will be used to categorise body mass

For peer review only - http://bmjopen.bmj.com/site/about/guidelines.xhtml

index: <18.5 kg/m^2, 18.5 to 24.9 kg/m^2, 25.0 to 29.9 kg/m^2, 30.0 to 34.9 kg/m^2, 35.0 to 39.9 kg/m^2, and 40+ kg/m^2.[35] Categories may be combined where numbers are too small.

(vi) Smoking

We shall include the latest available pre-pregnancy smoking status for the pregnant women. Smoking status will be categorised as: non-smoker, ex-smoker, and smoker.

(vii) Year (pregnancy start date)

Data quality and clinical guidelines may vary by year. Its effect on outcomes will be accounted for by adjusting for year of conception in the analysis.

Statistical analysis

We anticipate analyses will commence in June 2023 with study completion by June 2024. Baseline characteristics of the study population and outcomes will be described with summary statistics. Modified Poisson regression will be performed to estimate the relative risks of study outcomes. Cox regression will be performed for longer-term outcomes. The unit of analysis will be the pregnancy episode.

A federated analysis approach will be used as data governance arrangements do not allow pooling of the data across the four nations. Each dataset will be analysed separately following a common study protocol. A common data model will be established and implemented across the dataset, building on our previous work harmonising the phenome definitions for exposure conditions.[10] The effect sizes will be pooled using random-effects meta-analyses with inverse variance weighting for the primary care and secondary care datasets respectively.[36]

Where rare combinations of health conditions and outcomes may lead to identification of an individual or at the prespecified minimum count allowed by each data source, we will suppress the output.

For peer review only

For peer review only - http://bmjopen.bmj.com/site/about/guidelines.xhtml

Pregnant women with more than one pregnancy episode

An individual may have more than one pregnancy over the study period. The pregnancy episodes of the same woman will not be independent of each other. The severity of the exposure variable (pre-existing multiple long-term conditions) may increase in later pregnancy episodes as the pregnant women accumulates more long-term health conditions. If a woman had an adverse pregnancy outcome, she is more at risk of the same adverse outcome in subsequent pregnancy episodes. We shall account for this clustering effect of women with more than one pregnancy episode during the study period using the Generalised Estimating Equation in the regression analyses.

Multiple pregnancies

The main analysis will be limited to singleton pregnancies. Outcomes for pregnant women with multiple long-term conditions and multiple pregnancies (i.e. twins and higher order pregnancies) will be analysed as a separate cohort.

Missing data

Where exposure and outcome conditions are identified based on diagnostic codes, the absence of the code will be considered as an absence of the condition. The level and types of missingness of covariates will be reviewed and where appropriate will be addressed with representing missing data as a separate category or multiple imputation with chain equation (MICE). For variables required to compute an outcome, missing values will be imputed using MICE. Example of these variables include birthweight, gestational age and baby's sex to

For peer review only

determine preterm birth and small for gestational age. For each outcome, the statistical analyses will be performed on the imputed datasets and the estimates will be pooled with Rubin's rule.

Sensitivity analyses

We shall conduct sensitivity analyses using (i) complete case analysis, (ii) varying definitions of maternal multiple long-term conditions exposure using D'Arcy et al's core exposure set,[30] and (iii) in primiparous women. The latter is to account for the fact that some long-term conditions can arise from complications from a previous pregnancy.

Patient and public involvement

The research question was informed by discussions with our patient and public involvement (PPI) advisory group and our PPI co-investigators NM and RP.

The selection of outcomes are guided by our ongoing work developing a core outcome set for studies of pregnant women with multiple long-term conditions, where patients are key stakeholders.[33]

Our PPI advisory group and PPI co-investigators will be involved in interpreting the study findings, producing lay summaries and infographics, and disseminating the study findings through their network.

ETHICS AND DISSEMINATION

Ethics approval

For peer review only - http://bmjopen.bmj.com/site/about/guidelines.xhtml

CPRD: CPRD has broad National Research Ethics Service Committee ethics approval for purely observational research using the primary care data and established data linkages. The study has been reviewed and approved by CPRD's Independent Scientific Advisory Committee (reference: 20_181R).

SAIL: In accordance with UK Health Research Authority guidance, ethical approval is not mandatory for studies using only anonymised data. The study has been approved by SAIL Information Governance Review Panel.

Scotland dataset: The study has been approved by the National Health Service Scotland Public Benefit and Privacy Panel for Health and Social Care (HSC-PBPP) and The University Teaching and Research Ethics Committee (UTREC) from the University of St Andrews.

NIMATS: The study has been approved by the Honest Broker Service Governance Board.

Born in Bradford: Ethics approval was granted by Bradford National Health Service Research Ethics Committee (ref 07/H1302/112) for the Born in Bradford cohort.

The proposed study is purely observational and will use anonymised research data. The study will not involve participant recruitment. Therefore, consent to participate is not required.

Consent for publication

This is not applicable as the manuscript is a study protocol. In the proposed study, we will use de-identified study data, therefore consent for publication will not be required.

Dissemination

Study findings will be submitted for publications in peer reviewed journals and presented at key conferences for health and social care professionals involved in the care of pregnant women with multiple long-term conditions and their children. We will also organise

For peer review only - http://bmjopen.bmj.com/site/about/guidelines.xhtml

dissemination events to share our findings with the public, service users, clinicians and researchers.

DISCUSSION

MuM-PreDiCT is a consortium across all four nations of the UK studying multiple long-term conditions in pregnancy. As part of MuM-PreDiCT's program of work, we outlined the protocol for an observational study of maternal and child outcomes for pregnant women with multiple long-term conditions, using routine health records and a birth cohort in the UK.

Comparison with current literature

A recent systematic review found seven observational studies on the association of pre-pregnancy multiple long-term conditions with adverse maternal outcomes.[8] The review found that pre-pregnancy multiple long-term conditions were associated with severe maternal morbidity, hypertensive disorders of pregnancy, and acute health care use in the perinatal period.[8] Most studies were conducted in the United States.[8] Authors of the review commented that many studies included conditions arising in pregnancy in defining multiple long-term conditions, making it difficult to examine the impact of chronic conditions on maternal health.[8]

This proposed study will be based in the UK and will use a broad range of long-term conditions selected by women and clinicians to define multiple long-term conditions. Pregnancy related conditions and complications will be treated as study outcomes and will not be included in the exposure's definition. We will also study outcomes across all stages of pregnancy and outcomes for both women and their children.

For peer review only - http://bmjopen.bmj.com/site/about/guidelines.xhtml

Strengths and limitations

This proposed study will utilise routine health records from all four nations of the UK (England, Scotland, Wales and Northern Ireland). The available data sources consist of anonymised patient records from primary and secondary care, community prescription data, and maternity care data from routine booking appointments (first antenatal appointment offered universally and as the gateway to access maternity care in the UK).

Rich data will also be available from a birth cohort from Bradford, an ethnically diverse population in England. Beyond examining maternal outcomes, linked mother baby data and the birth cohort data will allow for the exploration of child outcomes. The key strength of this proposed study therefore is the generalisability of study findings to the UK population. Observing similar effect sizes across the different datasets will also increase the confidence in the study findings. Conversely, discrepancy in findings will stimulate further exploration of the datasets which may generate new knowledge.

As this is an observational study using anonymised routine health records, key limitations include missing data, misclassification bias due to inaccurate clinical coding and residual confounding.

Maternal multimorbidity will be quantified with simple counts. The severity of each health conditions will not be captured and the dose-response relationship will only be reflected in the total number of pre-existing long-term conditions. A systematic review of comorbidity indices used in maternal health research found three indices: Maternal Comorbidity Index, Charlson comorbidity index and Elixhauser comorbidity index.[27] Only the Maternal Comorbidity Index was developed from pregnant and postpartum women.[27] It was developed using hospital data with 20 maternal comorbidities but it included pregnancy related complications and factors such as multiple gestation, gestational diabetes, and hypertension

For peer review only - http://bmjopen.bmj.com/site/about/guidelines.xhtml

disorder of pregnancy.[27 28] In contrast, the list of health conditions we will use to define maternal pre-existing multimorbidity is more comprehensive and included leading causes of indirect maternal death (e.g. epilepsy) and mental health conditions.

Exposure and outcome events are only captured in routine health records when the pregnant women have presented to primary or secondary care and therefore the true prevalence and incidence may be underestimated. Health conditions that are managed conservatively in primary care, such as depression, anxiety and miscarriage, may not be captured in secondary care datasets. Events such as termination of pregnancy that occurred outside of the traditional health care settings may also be underestimated.[37] Similarly, antenatal hospital admission data may not reflect the full burden of additional antenatal appointments or acute care attendances, as care accessed through other routes may not be captured.

Body mass index, which encompasses underweight and obese categories, will be studied as a covariate instead of being counted as part of multimorbidity. There is much debate around whether obesity should be considered a disease[38] or a risk factor for other long-term conditions such as cardiometabolic conditions and cancers.[39-41] What is clear is pre-pregnancy maternal obesity is associated with adverse pregnancy outcome and dedicated care guideline has been established to manage this risk.[42 43] Studying body mass index as a separate variable will allow us to examine its independent effect and evidence may reaffirm its role as a modifiable risk factor for pregnant women with multiple long-term conditions.

Clinical implications

Current obstetric guidelines for pregnant women with medical conditions are focused on specific and single health conditions. There are currently no guidelines for the management of pregnant women with multiple long-term conditions in the UK. The heterogeneity of multiple long-term conditions means an all-encompassing guideline for every possible

For peer review only - http://bmjopen.bmj.com/site/about/guidelines.xhtml

combination of long-term conditions would not be possible. Indeed the English national guideline for multimorbidity focuses on general approaches such as coordinated and holistic care, improving quality of life by reducing treatment burden and shared decision making between patients and clinicians.[44] A guideline for multiple long-term conditions (multimorbidity) in pregnancy is likely to follow the same principles but with additional focus on the maternity care aspect.

The basis of shared decision making is the provision of evidence based information. As observed in the systematic review, there is currently a lack of evidence on the consequences of pregnancy for women with multiple long-term conditions.[8] Our PPI advisory group and preliminary findings from our core outcome set development work have highlighted how women valued having information to help them mentally prepare to face potential adverse pregnancy outcomes. The output from this study will therefore provide valuable information for women to make informed decision with their clinicians about family planning and their preconception, pregnancy and postpartum care. It will also provide valuable information to guide the future design of care pathway for women with multiple long-term conditions.

For peer review only

For peer review only - http://bmjopen.bmj.com/site/about/guidelines.xhtml

ABBREVIATIONS

CPRD	Clinical Practice Research Datalink
ICD-10	International Classification of Disease 10th version
MICE	Multiple imputation with chain equation
NICE	National Institute for Health and Care Excellence
NIMATS	Northern Ireland Maternity System
OPCS	Operating Procedures Codes
PPI	Patient and public involvement
SAIL	Secure Anonymised Information Linkage
UK	United Kingdom

For peer review only

For peer review only - http://bmjopen.bmj.com/site/about/guidelines.xhtml

Data availability statement

This is not applicable as the manuscript is a study protocol. In the proposed study, the data that support the findings are available from CPRD, SAIL, Scotland National Health Service Scotland Public Benefit and Privacy Panel for Health and Social Care, NIMATS and Born in Bradford, but restrictions apply to the availability of these data, which were used under license for the current study, and so are not publicly available. Data are however available from the authors upon reasonable request and with permission of CPRD, SAIL, Scotland National Health Service Scotland Public Benefit and Privacy Panel for Health and Social Care, NIMATS and Born in Bradford.

Competing interests

The authors declare that they have no competing interests.

Funding

This work is funded by the Strategic Priority Fund "Tackling multimorbidity at scale" programme (grant number MR/W014432/1) delivered by the Medical Research Council and the National Institute for Health Research (NIHR) in partnership with the Economic and Social Research Council and in collaboration with the Engineering and Physical Sciences Research Council. The views expressed are those of the author and not necessarily those of the funders, the NIHR or the UK Department of Health and Social Care. The funders had no role in study design, decision to publish, or preparation of the manuscript.

Contributors

SIL – Conceptualisation, funding acquisition, methodology, writing (original draft preparation).

KN, MB, KAE, KMA, DOR - Conceptualisation, funding acquisition, methodology, supervision, writing (review and editing)

For peer review only

For peer review only - http://bmjopen.bmj.com/site/about/guidelines.xhtml

HH, GS, AS, NM, AAL, AFF, CNP, CY, CMC, JIK, PB, RP, RR, ST, SB, UA, ZV- Conceptualisation, funding acquisition, methodology, writing (review and editing)

LK, KP, MS, MM, NC, SPBHS - Conceptualisation, methodology, writing (review and editing)

All authors read and approved the manuscript.

For peer review only

For peer review only - http://bmjopen.bmj.com/site/about/guidelines.xhtml

REFERENCES

1. Eggleton EJ, McMurrugh KJ, Aiken CE. Maternal pregnancy outcomes in women with cardiomyopathy: a systematic review and meta-analysis. *Am J Obstet Gynecol* 2022;227(4):582-92. doi: 10.1016/j.ajog.2022.05.039 [published Online First: 20220521]
2. Roos-Hesselink J, Baris L, Johnson M, et al. Pregnancy outcomes in women with cardiovascular disease: evolving trends over 10 years in the ESC Registry Of Pregnancy And Cardiac disease (ROPAC). *Eur Heart J* 2019;40(47):3848-55. doi: 10.1093/eurheartj/ehz136
3. Wiles K, Webster P, Seed PT, et al. The impact of chronic kidney disease Stages 3–5 on pregnancy outcomes. *Nephrology Dialysis Transplantation* 2020;36(11):2008-17. doi: 10.1093/ndt/gfaa247
4. Viale L, Allotey J, Cheong-See F, et al. Epilepsy in pregnancy and reproductive outcomes: a systematic review and meta-analysis. *Lancet (London, England)* 2015;386(10006):1845-52. doi: 10.1016/s0140-6736(15)00045-8 [published Online First: 20150825]
5. World Health Organization. Multimorbidity: Technical Series on Safer Primary Care. 2016. https://apps.who.int/iris/bitstream/handle/10665/252275/9789241511650-eng.pdf.
6. Admon LK, Winkelman TNA, Heisler M, et al. Obstetric Outcomes and Delivery-Related Health Care Utilization and Costs Among Pregnant Women With Multiple Chronic Conditions. *Preventing Chronic Disease* 2018;15:E21.
7. D'Arcy R, Knight M, Mackillop L. A retrospective audit of the socio-demographic characteristics and pregnancy outcomes for all women with multiple medical problems giving birth at a tertiary hospital in the UK in 2016. *BJOG: An International Journal of Obstetrics and Gynaecology* 2019;126:128.
8. Brown HK, McKnight A, Aker A. Association between pre-pregnancy multimorbidity and adverse maternal outcomes: A systematic review. *Journal of multimorbidity and comorbidity* 2022;12:26335565221096584. doi: 10.1177/26335565221096584 [published Online First: 2022/05/20]
9. Knight M, Bunch K, Tuffnell D, et al. Saving Lives, Improving Mothers' Care - Lessons learned to inform maternity care from the UK and Ireland Confidential Enquiries into Maternal Deaths and Morbidity 2016-18. Oxford: National Perinatal Epidemiology Unit, University of Oxford, 2020.
10. Lee SI, Azcoaga-Lorenzo A, Agrawal U, et al. Epidemiology of pre-existing multimorbidity in pregnant women in the UK in 2018: a population-based cross-sectional study. *BMC pregnancy and childbirth* 2022;22(1):120. doi: 10.1186/s12884-022-04442-3
11. Office for National Statistics. Childbearing for women born in different years, England and Wales: 2020. 2022 [cited 2022 20th December]. Available from: https://www.ons.gov.uk/peoplepopulationandcommunity/birthsdeathsandmarriages/conceptionandfertilityrates/bulletins/childbearingforwomenbornindifferentyearsenglandandwales/2020.
12. OECD Family Database. SF2.3: Age of mothers at childbirth and age-specific fertility. 2022. https://www.oecd.org/els/soc/SF_2_3_Age_mothers_childbirth.pdf.
13. Chen C, Xu X, Yan Y. Estimated global overweight and obesity burden in pregnant women based on panel data model. *PLoS One* 2018;13(8):e0202183. doi: 10.1371/journal.pone.0202183 [published Online First: 20180809]
14. Wang MC, Freaney PM, Perak AM, et al. Trends in Prepregnancy Obesity and Association With Adverse Pregnancy Outcomes in the United States, 2013 to 2018. *J Am Heart Assoc* 2021;10(17):e020717. doi: 10.1161/jaha.120.020717 [published Online First: 20210825]
15. Beeson JG, Homer CSE, Morgan C, et al. Multiple morbidities in pregnancy: Time for research, innovation, and action. *PLoS medicine* 2018;15(9):e1002665. doi: 10.1371/journal.pmed.1002665 [published Online First: 2018/09/27]

For peer review only - http://bmjopen.bmj.com/site/about/guidelines.xhtml

16. Herrett E, Gallagher AM, Bhaskaran K, et al. Data Resource Profile: Clinical Practice Research Datalink (CPRD). *International Journal of Epidemiology* 2015;44(3):827-36. doi: 10.1093/ije/dyv098
17. National Institute for Health and Care Excellence. Clinical Knowledge Summaries: Antenatal Care - Uncomplicated Pregnancy. 2021 [Available from: https://cks.nice.org.uk/topics/antenatal-care-uncomplicated-pregnancy/management/antenatal-care-uncomplicated-pregnancy/#booking-appointment accessed 5th July 2022.
18. Hatherall B, Morris J, Jamal F, et al. Timing of the initiation of antenatal care: An exploratory qualitative study of women and service providers in East London. *Midwifery* 2016;36:1-7. doi: 10.1016/j.midw.2016.02.017 [published Online First: 2016/04/24]
19. Office for National Statistics. Birth characteristics in England and Wales: 2020. 2022 [Available from: https://www.ons.gov.uk/peoplepopulationandcommunity/birthsdeathsandmarriages/livebirths/bulletins/birthcharacteristicsinenglandandwales/2020#place-of-birth accessed 12th July 2022.
20. Raynor P. Born in Bradford, a cohort study of babies born in Bradford, and their parents: protocol for the recruitment phase. *BMC public health* 2008;8:327. doi: 10.1186/1471-2458-8-327 [published Online First: 2008/09/25]
21. CPRD. Release notes: CPRD GOLD May 2022. 2022 [Available from: https://cprd.com/sites/default/files/2022-05/2022-05%20CPRD%20GOLD%20Release%20Notes.pdf accessed 14th July 2022.
22. SAIL Databank. 10 years of spearheading data privacy and research utility [cited 2021 16th February]. Available from: https://saildatabank.com/wp-content/uploads/SAIL_10_year_anniversary_brochure.pdf.
23. Health Data Research Innovation Gateway. Northern Ireland Regional Maternity system (NIMATS) 2021 [cited 2021 7 December]. Available from: https://web.www.healthdatagateway.org/dataset/89d76819-32cb-48ad-9337-880f324a6ec0.
24. Born in Bradford. About Us 2021 [cited 2021 7 December]. Available from: https://borninbradford.nhs.uk/about-us/.
25. Ho IS, Azcoaga-Lorenzo A, Akbari A, et al. Variation in the estimated prevalence of multimorbidity: systematic review and meta-analysis of 193 international studies. *BMJ Open* 2022;12(4):e057017. doi: 10.1136/bmjopen-2021-057017 [published Online First: 20220429]
26. Ho IS-S, Azcoaga-Lorenzo A, Akbari A, et al. Examining variation in the measurement of multimorbidity in research: a systematic review of 566 studies. *The Lancet Public Health* 2021;6(8):e587-e97. doi: 10.1016/S2468-2667(21)00107-9
27. Aoyama K, D'Souza R, Inada E, et al. Measurement properties of comorbidity indices in maternal health research: a systematic review. *BMC Pregnancy & Childbirth* 2017;17(1):372.
28. Bateman BT, Mhyre JM, Hernandez-Diaz S, et al. Development of a comorbidity index for use in obstetric patients. *Obstetrics and Gynecology* 2013;122(5):957-65. doi: 10.1097/AOG.0b013e3182a603bb
29. Kuan V, Denaxas S, Gonzalez-Izquierdo A, et al. A chronological map of 308 physical and mental health conditions from 4 million individuals in the English National Health Service. *Lancet Digit Health* 2019;1(2):e63-e77. doi: 10.1016/s2589-7500(19)30012-3 [published Online First: 20190520]
30. D'Arcy R, Knight M. List of health conditions to define multiple long-term conditions in pregnancy. [Available from: https://drive.google.com/file/d/1Q3LTzcXGnEFcS6eMAA0pntEUUUa-iTtp/view accessed 14th July 2022.

For peer review only - http://bmjopen.bmj.com/site/about/guidelines.xhtml

31. Devane D, Begley CM, Clarke M, et al. Evaluating maternity care: a core set of outcome measures. *Birth (Berkeley, Calif)* 2007;34(2):164-72. doi: 10.1111/j.1523-536X.2006.00145.x [published Online First: 2007/06/05]
32. Nijagal MA, Wissig S, Stowell C, et al. Standardized outcome measures for pregnancy and childbirth, an ICHOM proposal. *BMC Health Services Research* 2018;18(1):953. doi: 10.1186/s12913-018-3732-3
33. Lee SI, Eastwood K-A, Moss N, et al. Protocol for the development of a core outcome set for studies of pregnant women with pre-existing multimorbidity. *BMJ Open* 2021;11(10):e044919. doi: 10.1136/bmjopen-2020-044919
34. World Health Organization. WHO Technical Consultation on Postpartum and Postnatal Care,. 2010. https://www.ncbi.nlm.nih.gov/books/NBK310595/ (accessed 6th July 2022).
35. World Health Organization. Obesity: preventing and managing the global epidemic. Report of a WHO consultation. *World Health Organization technical report series* 2000;894:i-xii, 1-253. [published Online First: 2001/03/10]
36. Borenstein M, Hedges LV, Higgins JP, et al. A basic introduction to fixed-effect and random-effects models for meta-analysis. *Res Synth Methods* 2010;1(2):97-111. doi: 10.1002/jrsm.12 [published Online First: 20101121]
37. Minassian C, Williams R, Meeraus WH, et al. Methods to generate and validate a Pregnancy Register in the UK Clinical Practice Research Datalink primary care database. *Pharmacoepidemiology and Drug Safety* 2019;28(7):923-33. doi: https://doi.org/10.1002/pds.4811
38. Burki T. European Commission classifies obesity as a chronic disease. *The Lancet Diabetes & Endocrinology* 2021;9(7):418. doi: 10.1016/S2213-8587(21)00145-5
39. Abdullah A, Peeters A, de Courten M, et al. The magnitude of association between overweight and obesity and the risk of diabetes: a meta-analysis of prospective cohort studies. *Diabetes Res Clin Pract* 2010;89(3):309-19. doi: 10.1016/j.diabres.2010.04.012 [published Online First: 20100520]
40. Bhaskaran K, Douglas I, Forbes H, et al. Body-mass index and risk of 22 specific cancers: a population-based cohort study of 5.24 million UK adults. *The Lancet* 2014;384(9945):755-65. doi: 10.1016/S0140-6736(14)60892-8
41. Powell-Wiley TM, Poirier P, Burke LE, et al. Obesity and Cardiovascular Disease: A Scientific Statement From the American Heart Association. *Circulation* 2021;143(21):e984-e1010. doi: doi:10.1161/CIR.0000000000000973
42. D'Souza R, Horyn I, Pavalagantharajah S, et al. Maternal body mass index and pregnancy outcomes: a systematic review and metaanalysis. *American Journal of Obstetrics & Gynecology MFM* 2019;1(4) doi: 10.1016/j.ajogmf.2019.100041
43. Royal College of Obstetricians & Gynaecologists. Care of Women with Obesity in Pregnancy (Green-top Guideline No. 72) 2018 [cited 2023 3rd January]. Available from: https://www.rcog.org.uk/guidance/browse-all-guidance/green-top-guidelines/care-of-women-with-obesity-in-pregnancy-green-top-guideline-no-72/.
44. National Institute for Health and Care Excellence. NICE guideline [NG56]: Multimorbidity: clinical assessment and management. 2016. https://www.nice.org.uk/guidance/ng56/resources/multimorbidity-clinical-assessment-and-management-pdf-1837516654789.

For peer review only - http://bmjopen.bmj.com/site/about/guidelines.xhtml

BMJ Open

Maternal and child outcomes for pregnant women with pre-existing multiple long-term conditions: protocol for an observational study in the United Kingdom

Journal:	*BMJ Open*
Manuscript ID	bmjopen-2022-068718.R2
Article Type:	Protocol
Date Submitted by the Author:	31-Jan-2023
Complete List of Authors:	Lee, Siang Ing; University of Birmingham Institute of Applied Health Research<br>Hope, Holly; The University of Manchester, Centre for Women's Mental Health, Faculty of Biology Medicine & Health<br>OReilly, Dermot; Queen's University Belfast, Centre for Public Health<br>Kent, Lisa; Queen's University Belfast, Centre for Public Health<br>Santorelli, Gillian; Bradford Institute for Health Research<br>Subramanian, Anuradhaa; University of Birmingham Institute of Applied Health Research<br>Moss, Ngawai; Patient and public representative<br>Azcoaga-Lorenzo, Amaya; University of St Andrews School of Medicine, Division of Population and Behavioural Sciences; Hospital Rey Juan Carlos, Instituto de Investigación Sanitaria Fundación Jimenez Diaz<br>Fagbamigbe, Adeniyi; University of St Andrews School of Medicine, Division of Population and Behavioural Sciences; University of Ibadan College of Medicine, Department of Epidemiology and Medical Statistics<br>Nelson-Piercy, Catherine; Guy's and St Thomas' NHS Foundation Trust<br>Yau, Christopher; University of Oxford, Nuffield Department of Women's and Reproductive Health; Health Data Research UK<br>McCowan, Colin ; University of St Andrews School of Medicine, Division of Population and Behavioural Sciences<br>Kennedy, Jonathan; Swansea University, Data Science, Medical School<br>Phillips, Katherine; University of Birmingham Institute of Applied Health Research<br>Singh, Megha; University of Birmingham Institute of Applied Health Research<br>Mhereeg, Mohamed; Swansea University, Data Science, Medical School<br>Cockburn, Neil; University of Birmingham Institute of Applied Health Research<br>Brocklehurst, Peter; University of Birmingham Institute of Applied Health Research<br>Plachcinski, Rachel; Patient and public representative<br>Riley, Richard; University of Birmingham Institute of Applied Health Research<br>Thangaratinam, Shakila; University of Birmingham Institute of Metabolism and Systems Research, WHO Collaborating Centre for Global Women's Health; Birmingham Women's and Children's NHS Foundation Trust, Department of Obstetrics and Gynaecology<br>Brophy, Sinead; Swansea University, Data Science, Medical School

	Hemali Sudasinghe, Sudasing Pathirannehelage Buddhika; University of Birmingham Institute of Applied Health Research<br>Agrawal, Utkarsh; University of St Andrews School of Medicine, Division of Population and Behavioural Sciences<br>Vowles, Zoe; Guy's and St Thomas' NHS Foundation Trust<br>Abel, Kathryn ; The University of Manchester, Centre for Women's Mental Health, Faculty of Biology Medicine & Health; Greater Manchester Mental Health NHS Foundation Trust<br>Nirantharakumar, Krishnarajah; University of Birmingham Institute of Applied Health Research<br>Black, Mairead; University of Aberdeen School of Medicine Medical Sciences and Nutrition, Aberdeen Centre for Women’s Health Research<br>Eastwood, Kelly-Ann; Queen's University Belfast, Centre for Public Health; University Hospitals Bristol and Weston NHS Foundation Trust<br>-, on behalf of MuM-PreDiCT; MuM-PreDiCT
**Primary Subject Heading**:	Obstetrics and gynaecology
Secondary Subject Heading:	Epidemiology
Keywords:	Maternal medicine < OBSTETRICS, OBSTETRICS, EPIDEMIOLOGY

SCHOLARONE™
Manuscripts

Maternal and child outcomes for pregnant women with pre-existing multiple long-term conditions: protocol for an observational study in the United Kingdom

Authors:

Siang Ing Lee,[1] Holly Hope,[2] Dermot O'Reilly,[3] Lisa Kent,[3] Gillian Santorelli,[4] Anuradhaa Subramanian,[1] Ngawai Moss,[5] Amaya Azcoaga-Lorenzo,[6, 7] Adeniyi Francis Fagbamigbe,[6, 8] Catherine Nelson-Piercy,[9] Christopher Yau,[10,11] Colin McCowan,[6] Jonathan I Kennedy,[12] Katherine Phillips,[1] Megha Singh,[1] Mohamed Mhereeg,[12] Neil Cockburn,[1] Peter Brocklehurst,[1] Rachel Plachcinski,[5] Richard Riley,[1] Shakila Thangaratinam,[13, 14] Sinead Brophy,[12] Sudasing Pathirannehelage Buddhika Hemali Sudasinghe,[1] Utkarsh Agrawal,[6] Zoe Vowles,[9] Kathryn M Abel,[2, 15] * Krishnarajah Nirantharakumar,[1]* Mairead Black,[16]* Kelly-Ann Eastwood,[3, 17]* on behalf of the MuM-PreDiCT Group

*Joint senior authors

Affiliations:

1. Institute of Applied Health Research, University of Birmingham, Birmingham, UK
2. Centre for Women's Mental Health, Faculty of Biology Medicine & Health, The University of Manchester, UK
3. Centre for Public Health, Queen's University of Belfast, UK
4. Bradford Institute for Health Research, UK
5. Patient and public representative, UK
6. Division of Population and Behavioural Sciences, School of Medicine, University of St Andrews, UK
7. Hospital Rey Juan Carlos. Instituto de Investigación Sanitaria Fundación Jimenez Diaz. Madrid. Spain
8. Department of Epidemiology and Medical Statistics, College of Medicine, University of Ibadan, Nigeria
9. Guy's and St. Thomas' NHS Foundation Trust, UK
10. Nuffield Department of Women's and Reproductive Health, University of Oxford, UK
11. Health Data Research UK
12. Data Science, Medical School, Swansea University, UK
13. WHO Collaborating Centre for Global Women's Health, Institute of Metabolism and Systems Research, University of Birmingham, UK
14. Department of Obstetrics and Gynaecology, Birmingham Women's and Children's NHS Foundation Trust, UK
15. Greater Manchester Mental Health NHS Foundation Trust, Manchester, UK
16. Aberdeen Centre for Women's Health Research, School of Medicine, Medical Science and Nutrition, University of Aberdeen, UK

For peer review only

17. St Michael's Hospital, University Hospitals Bristol NHS Foundation Trust, UK

Correspondence to:

Krishnarajah Nirantharakumar

K.Nirantharan@bham.ac.uk

Authors' ORCID:

Siang Ing Lee 0000-0002-2332-5452, Holly Hope 0000-0002-4834-6719, Dermot O'Reilly 0000-0002-9181-0652, Lisa Kent 0000-0002-8882-0526, Gillian Santorelli 0000-0003-0427-1783, Anuradhaa Subramanian 0000-0001-8875-7363, Ngawai Moss 0000-0001-9369-5072, Amaya Azcoaga-Lorenzo 0000-0003-3307-878X, Adeniyi Francis Fagbamigbe 0000-0001-9184-8258, Catherine Nelson-Piercy 0000-0001-9311-1196, Christopher Yau 0000-0001-7615-8523, Colin McCowan 0000-0002-9466-833X, Jonathan I Kennedy 0000-0002-1122-6502, Katherine Phillips 0000-0003-0674-605X, Megha Singh 0000-0003-3680-7124, Mohamed Mhereeg 0000-0003-1241-9549, Neil Cockburn 0000-0001-9284-6991, Peter Brocklehurst 0000-0002-9950-6751, Rachel Plachcinski 0000-0001-9908-0773, Richard Riley 0000-0001-8699-0735, Shakila Thangaratinam 0000-0002-4254-460X, Sinead Brophy 0000-0001-7417-2858, Utkarsh Agrawal 0000-0001-5181-6120, Zoe Vowles 0000-0001-6989-2180, Kathryn M Abel 0000-0003-3538-8896, Krishnarajah Nirantharakumar 0000-0002-6816-1279, Mairead Black 0000-0002-6841-8601, Kelly-Ann Eastwood 0000-0003-3689-0490.

Main text word count: 3965

Keywords: multimorbidity, multiple chronic conditions, multiple long-term conditions, pregnancy, maternity, obstetric, outcome, children, offspring

iew only

For peer review only - http://bmjopen.bmj.com/site/about/guidelines.xhtml

ABSTRACT

Introduction: One in five pregnant women have multiple pre-existing long-term conditions in the United Kingdom (UK). Studies have shown that maternal multiple long-term conditions are associated with adverse outcomes. This observational study aims to compare maternal and child outcomes for pregnant women with multiple long-term conditions to those without multiple long-term conditions (0 or 1 long-term conditions).

Methods and analysis: Pregnant women aged 15 to 49 years old with a conception date between 2000 and 2019 in the UK will be included with follow up till 2019. The data source will be routine health records from all four UK nations (Clinical Practice Research Datalink [CPRD, England], Secure Anonymised Information Linkage [SAIL, Wales], Scotland routine health records and Northern Ireland Maternity System [NIMATS]); and the Born in Bradford birth cohort. The exposure of two or more pre-existing, long-term physical or mental health conditions will be defined from a list of health conditions predetermined by women and clinicians. The association of maternal multiple long-term conditions with (i) antenatal, (ii) peripartum, (iii) postnatal and long-term, and (iv) mental health outcomes, for both women and their children will be examined. Outcomes of interest will be guided by a core outcome set. Comparisons will be made between pregnant women with and without multiple long-term conditions using modified Poisson and Cox regression. Generalised estimating equation will account for the clustering effect of women who had more than one pregnancy episode. Where appropriate, multiple imputation with chained equation will be used for missing data. Federated analysis will be conducted for each dataset and results will be pooled using random-effects meta-analyses.

Ethics and dissemination: Approval has been obtained from the respective data sources in each UK nation. Study findings will be submitted for publications in peer reviewed journals and presented at key conferences.

For peer review only

ARTICLE SUMMARY

Strengths and limitations of this study

- The study will utilise rich data sources from routine health records from all four UK nations and a birth cohort.
- Beyond examining maternal outcomes, linked mother baby data and the birth cohort data will allow for the exploration of children's outcomes.
- Key limitations include missing data, misclassification bias due to inaccurate clinical coding and residual confounding.

For peer review only

For peer review only - http://bmjopen.bmj.com/site/about/guidelines.xhtml

INTRODUCTION

Maternal single long-term conditions such as cardiac conditions, chronic kidney disease and epilepsy are associated with adverse pregnancy outcomes.[1-4] This is likely to be compounded when the pregnant woman has two or more long-term physical or mental health conditions (multimorbidity). Some conditions may need different treatments from different health care teams, thereby increasing the treatment burden and complexity of care.[5] Recent evidence has shown that maternal multiple long-term conditions are associated with adverse outcomes for women and their children, such as severe maternal morbidity and mortality, pre-eclampsia, emergency caesarean birth, preterm birth, and low birth weight.[6-8] In the UK 2016-18 national maternal mortality report, 90% of women who died during or up to a year after pregnancy had multiple health or social problems.[9]

Currently one in five pregnant women have multiple long-term conditions prior to pregnancy in the United Kingdom (UK).[10] The number of pregnant women with pre-existing multiple long-term conditions is likely to increase as women are getting pregnant later in life and with higher body weight.[11-14] As this becomes an increasingly important issue, information on pregnancy, maternal and child outcomes is crucial for women and their health care professionals to make informed decisions on preconception and pregnancy care planning. However, there remains a lack of evidence to guide care pathways for pregnant women with multiple long-term conditions.[8 15]

Healthcare is free in the UK and over 98% of the population are registered at a general practice (akin to family practice in other countries).[16] General practices not only provide primary and community healthcare, but they also serve as the main point of contact for referrals to specialist clinical services and provide the majority of prescribing outside of a hospital setting.[16] In the UK, pregnant women are recommended to have their booking

For peer review only - http://bmjopen.bmj.com/site/about/guidelines.xhtml

124 appointment before 10 weeks gestation.[17] This is the pregnant woman's first midwife or doctor appointment, where they undergo health and social care assessment of needs and risks for her pregnancy.[18] Over 97% of births occur in healthcare settings in England and Wales.[19] Therefore, routine health records in primary and secondary care in the UK offer a rich data source for observational studies of pregnant women and their children.

129 This observational study aims to compare outcomes for women with multiple long-term conditions to those without multiple long-term conditions. Outcomes studied will include those for women and their children. Datasets from routine health records from all four UK nations (England, Wales, Scotland, and Northern Ireland) will be used. In addition, the Born in Bradford birth cohort from a deprived, ethnically diverse city in the UK, will also be used.[20]

135 The four research objectives are to examine the association between maternal pre-existing multiple long-term conditions with: (1) antenatal, (2) peripartum, (3) postnatal and long-term outcomes, and (4) mental health outcomes. The findings from each research objective will be published in a separate paper.

For peer review only - http://bmjopen.bmj.com/site/about/guidelines.xhtml

METHODS AND ANALYSIS

Study design

This is a cohort observational study using data from routine healthcare records and a birth cohort in the UK.

Study population and eligibility criteria

The study population will consist of women aged 15-49 years old at conception, with pregnancies beginning between 1st January 2000 and 31st December 2019 in the UK. Date of conception (pregnancy start date) will be defined as the first day of the last menstrual period or gestational day 0. To ensure sufficient quality data, eligible women must have health records that meet the standard data quality checks as defined by each data source and one year's worth of health records prior to index pregnancy.

Data sources

Table 1 presents the five data sources that will be used. Each UK devolved nation is represented by a population based routine health record dataset, with good national coverage for Wales, Scotland and Northern Ireland and a representative sample for England.[16] The exposure status will be determined from primary care records for Clinical Practice Research Datalink (CPRD) and Secure Anonymised Information Linkage (SAIL), with CPRD GOLD representing 5% of UK general practices,[21] and SAIL covering 80% of Welsh general practices.[22] For Scotland's linked routine records and Northern Ireland Maternity System (NIMATS), the exposure status will be determined from hospital and prescribing records.

For peer review only - http://bmjopen.bmj.com/site/about/guidelines.xhtml

163 CPRD and SAIL's primary care data offer the opportunity to study outcomes that may not be captured in secondary care. For instance, vomiting in pregnancy, miscarriage and neurodevelopmental conditions in children. The Scottish dataset provides detailed information on the different types of hospital attendances, including psychiatric admissions and accident and emergency attendances. NIMATS's unique first antenatal visit dataset is a good source of pre-pregnancy clinical data not available in other datasets.

169 As routine health records were not collected for research purposes, it is prone to missing data. Therefore, we have also included Born in Bradford, a regional birth cohort (2007-2011) where data were collected systematically and longitudinally from pregnancy, childhood through to adult life.

For peer review only

For peer review only - http://bmjopen.bmj.com/site/about/guidelines.xhtml

Table 1. Summary of data sources

Name of data source	Country	Population: pregnant women	Exposure: maternal multiple long-term conditions status	Outcomes: pregnant women	Outcomes: children
Clinical Practice Research Datalink (CPRD)[16]	England	Pregnancy register (primary care)	Primary care routine health records	Primary care records, hospital admissions, death registration	Mother-baby linked data: primary care records, hospital admissions, death registration
Secure Anonymised Information Linkage (SAIL)[22]	Wales	Births from National Community Child Health Dataset	Primary care routine health records	Primary care records, hospital admissions, death registration	Mother-baby linked data: primary care records, hospital admissions, death registration
Scotland routine health records	Scotland	Scottish Maternity Records, pregnancy-related hospital admissions	Hospital admissions, psychiatric admissions, accident and emergency attendances, prescriptions	Hospital admissions, psychiatric admissions, accident and emergency attendances, death registration	Mother-baby linked data: hospital admissions, psychiatric admissions, accident and emergency attendances, death registration
Northern Ireland Maternity System (NIMATS)[23]	Northern Ireland	Maternity booking (first antenatal) appointment records, birth related hospital admissions	Maternity booking (first antenatal) appointment records, birth related hospital admissions, prescriptions	Hospital admissions	Mother-baby linked data: hospital admissions
Born in Bradford[24]	Bradford, England	Birth cohort of over 13,500 children born from around 12,500 mothers at the Bradford Royal Infirmary between March 2007 and June 2011	Primary care routine health records	Data from birth cohort: clinical data<br>Data from linked health records: maternity, primary care, hospital admissions	Data from birth cohort: offspring developmental, clinical and education data<br>Data from linked health records: primary care, hospital admissions

For peer review only - http://bmjopen.bmj.com/site/about/guidelines.xhtml

Exposure

The exposed group will consist of pregnant women with multiple long-term conditions. Measurements of multiple long-term conditions are variable in existing literature.[25 26] Currently only Bateman et al's Maternal Comorbidity Index has been developed specifically for obstetric research.[27 28] It consists of 20 health conditions and included conditions arising in pregnancy such as gestational hypertension, pre-eclampsia and placenta praevia.[28] This limits the ability to study the impact of pre-existing long-term conditions on maternal and child health and the implication for long-term condition management preconception.[8]

In this study, we shall define multiple long-term conditions as two or more long-term physical or mental health conditions that pre-existed before pregnancy. Pregnancy related complications will not be included as they will be studied as outcomes. Multiple long-term conditions will be defined from a list of 79 health conditions previously described in our epidemiological work (Table 2) and will be measured with simple count.[10] This list was compiled from existing multimorbidity literature[9 26 29] and a workshop with our multidisciplinary research advisory group, including patient representatives and clinicians.[10] Selection of health conditions were based on: (i) prevalence; (ii) potential to impact on pregnancy outcomes; (iii) considered important by women; and (iv) recorded in the study datasets.[10] The phenome definitions for these health conditions have previously been described in our epidemiological work.[10] For health conditions that are transient and episodic in nature (e.g. asthma, eczema, depression and anxiety), we will only include the condition if it is active, which we have defined as requiring a doctors' consultation or medical prescription in the 12 months preceding pregnancy.[10] Sensitivity analysis will be performed defining maternal multiple long-term conditions with a different list of health conditions by D'Arcy et al.[30]

For peer review only - http://bmjopen.bmj.com/site/about/guidelines.xhtml

Exposure will be ascertained by the presence of diagnostic or prescriptions codes, including Read (to identify exposures in primary care data) and International Classification of Disease 10th version (ICD-10, secondary care).

Table 2. List of 79 health conditions defining multiple long-term conditions in pregnancy

Cancers

1. All cancers
    - Solid cancers
    - Haematological cancers
    - Metastatic cancers
    - Exclude basal cell carcinoma

Cardiovascular disease

2. Hypertension
3. Ischemic heart disease & myocardial infarction
4. Heart failure
5. Stroke
    - Transient ischemic attack
    - Ischemic stroke
    - Haemorrhagic stroke
    - Unspecified stroke
6. Atrial fibrillation
7. Congenital heart disease
8. Valvular heart disease (mitral, aortic, mixed)
9. Cardiomyopathy

Dermatology

10. Eczema
11. Psoriasis
12. Autoimmune skin disease
    - Vitiligo
    - Alopecia areata
13. Other dermatological conditions
    - Seborrheic dermatitis
    - Rosacea
    - Hidradenitis suppurativa
    - Lichen planus

Ear, Nose, Throat

14. Profound deafness
15. Allergic rhinitis & allergic conjunctivitis

Eye

16. Inflammatory eye disease
    - Scleritis & episcleritis
    - Anterior uveitis
    - Posterior uveitis
17. Cataract
18. Diabetic eye disease
19. Severe blindness
20. Retinal detachment

Neurodevelopmental conditions

43. Neurodevelopmental conditions
    - Learning disability
    - Attention deficit hyperactivity disorder
    - Autistic spectrum disorder

Rheumatology

44. Systemic lupus erythematosus
45. Spondylarthritis
    - Psoriatic arthritis
    - Ankylosing spondylitis
46. Inflammatory arthritis
    - Rheumatoid arthritis
    - Sjogern's syndrome
    - Raynaud's syndrome
    - Systemic sclerosis
    - Primary systemic vasculitis
47. Ehler's Danlos Syndrome (EDS) Type 3 (Hypermobile EDS)

Orthopaedic

48. Scoliosis
49. Vertebral disorder
    - Intervertebral disc disorder
    - Spondylosis
    - Spondylolisthesis
    - Collapsed vertebrae
    - Spinal stenosis
50. Chronic back pain
51. Osteoporosis
52. Osteoarthritis

Neurology

53. Migraine
54. Other chronic headache (including cluster headache, tension headache)
55. Epilepsy
56. Multiple sclerosis
57. Spina bifida
58. Idiopathic intracranial hypertension
59. Peripheral neuropathy
60. Other neurological conditions / musculoskeletal disorders
    - chronic fatigue syndrome / myalgic encephalomyelitis
    - fibromyalgia

For peer review only - http://bmjopen.bmj.com/site/about/guidelines.xhtml

Gastroenterology

21. Irritable bowel syndrome
22. Inflammatory bowel disease
    - Ulcerative colitis
    - Crohn's disease
23. Coeliac disease
24. Chronic liver disease
    - Chronic hepatitis B & C
    - Alcoholic liver disease
    - Autoimmune liver disease
    - Cirrhosis
    - Non-alcoholic fatty liver disease
25. Peptic ulcer
26. Gall stones

Gynaecology

27. Polycystic ovarian syndrome
28. Endometriosis
29. Fibroids
30. Infertility

Haematology

31. History of venous thromboembolism
    - Deep vein thrombosis
    - Pulmonary embolism
32. Primary thrombocytopenia
33. Haemophilia
34. Sickle cell anaemia
35. Pernicious anaemia

Mental health

36. Depression
37. Anxiety
    - Panic disorder
    - Phobia disorder
    - Post-traumatic stress disorder
38. Severe mental illness
    - Bipolar affective disorder
    - Schizophrenia
    - Psychosis
39. Eating disorder
40. History of alcohol use disorder (misuse / dependence)
41. History of substance misuse
42. Others
    - Obsessive compulsive disorder
    - Self-harm
    - Personality disorder
    - Dissociative disorder

- chronic pain syndrome (includes chronic regional pain syndrome, myofascial pain syndrome)

Respiratory

61. Asthma
62. Chronic obstructive pulmonary disease
63. Obstructive sleep apnoea
64. Pulmonary fibrosis, interstitial lung disease
65. Pulmonary hypertension
66. Bronchiectasis
67. Cystic fibrosis
68. Sarcoidosis

Renal

69. Chronic kidney disease
70. Urinary tract stones

Endocrine

71. Diabetes mellitus
72. Thyroid disorder
73. Pituitary disorder
74. Adrenal benign tumour
75. Hyperparathyroidism

Other

76. Human immunodeficiency viral infection / Acquired immune deficiency syndrome
77. Turner's syndrome
78. Marfan's syndrome
79. Solid organ transplant

For peer review only

Comparator

Multiple long-term conditions versus no multiple long-term conditions

Comparisons will be made with the following exposure group:

For peer review only - http://bmjopen.bmj.com/site/about/guidelines.xhtml

(i) pregnant women with multiple long-term conditions;

(ii) pregnant women with increasing counts of long-term health conditions;

(iii) pregnant women with different combinations of long-term health conditions; and

(iv) pregnant women in different health condition clusters (identified from ongoing clustering analyses).

The selection of which combinations and clusters of long-term conditions to study will be based on how common they are and their clinical relevance, following consultation with patient representatives and clinicians in our research team. Pregnant women with no multiple long-term conditions (i.e. no or single long-term conditions) will be the common comparator group.

Multiple long-term conditions with and without mental illness

In addition, we will also compare the outcomes for pregnant women who have mental health conditions as part of their multiple long-term conditions against pregnant women with multiple long-term conditions who do not have mental health conditions.

Outcomes

The outcomes will be grouped into the following four categories based on the research objectives: (1) antenatal, (2) peripartum, (3) postnatal and long-term outcomes, and (4) mental health outcomes. Examples of outcomes are provided as follows, based on existing core outcome sets for pregnancy and childbirth.[31 32] The definitive list of outcomes will be confirmed once the development work for a core outcome set for studies of pregnant women with multiple long-term conditions is completed.[33] Outcomes will be ascertained from the study datasets (1st January 2000 to 31st December 2019) using clinical codes, such as Read,

For peer review only - http://bmjopen.bmj.com/site/about/guidelines.xhtml

ICD-10 and Operating Procedures Codes (OPCS) Classification of Interventions and Procedures.

(1) Antenatal

Antenatal outcomes occur from conception to before the onset of childbirth. Examples for women include miscarriage, gestational hypertension, pre-eclampsia, gestational diabetes, venous thromboembolism, placenta abruption and antenatal hospital admissions. Examples for children include fetal growth restriction.

(2) Peripartum

Peripartum outcomes occur during and immediately after childbirth. This category will also include survival outcomes for women and children. Examples for women include mode of birth (spontaneous vaginal birth , birth with forceps/ ventouse, caesarean birth), postpartum haemorrhage, severe maternal morbidity, admission to intensive care and maternal death. Examples for children include preterm birth, small for gestational age, admission to neonatal unit, stillbirth, perinatal death and neonatal death.

(3) Postnatal and long-term

Postnatal outcomes occur in the 42 days after birth,[34] while long-term outcomes are beyond the peripartum and postpartum period. For women this would include functional outcomes such as incontinence. For children, we will use mother baby linked primary and secondary care data to study postnatal and long-term outcomes such as congenital anomalies, neurodevelopmental conditions (e.g. autism, attention deficit hyperactive disorder and learning difficulty), cerebral palsy, and chronic lung disease. The length of follow up will depend on the availability of data in the routine health records. For example, CPRD has a median follow up of 5 years.[16] We will also examine postpartum readmission for mother and child.

For peer review only - http://bmjopen.bmj.com/site/about/guidelines.xhtml

(4) Mental health

Mental health outcomes cover the antenatal and postnatal period and will be considered up to 12 months after birth. This is to account for possible delay in women presenting to clinicians and reaching a formal diagnosis. We will consider both: (i) incident and (ii) recurrent mental health outcomes, where incident means a woman enters the analysis with no prior record of the specific mental health outcome. A perinatal mental health event is indicated by a primary care visit or hospital admission and includes mental health outcomes of concern in the antenatal and postnatal period (e.g.depression, psychosis, post-traumatic stress disorder, self-harm and suicide attempts). Comparing the mental health event rates of pregnant women who have and have not got mental health conditions as part of their multiple long-term conditions will allow us to delineate the contribution of mental and physical morbidity to perinatal mental health outcomes. Children's mental ill health will also be considered (e.g. depression and anxiety).

Covariates

Analyses will adjust for the following covariates. Additional covariates may be added for individual outcomes based on the literature. For example, in analyses of mental health outcomes there will be additional covariates. For the mother, we will include history of any mental illness, for the child we will include maternal history of any mental and/ or neurodevelopmental conditions.

Where data for antenatal exposures are available (e.g. from NIMATS and Born in Bradford's booking appointments), additional analyses may be conducted where appropriate.

(i) Maternal age

For peer review only

We shall explore whether the association between maternal age and the outcomes are linear. Where this is not the case and to aid clinical interpretability, we will categorise maternal age at conception into 5-yearly age bands.

(ii) Parity/gravidity

The variable used will depend on availability in study datasets. Where both variables are available, both will be reported with preference given to *parity (*the number of times a woman gave birth at gestation ≥24 weeks); and sensitivity analysis will be conducted using *gravidity* (the number of times a woman has been pregnant).

(iii) Ethnicity

Maternal ethnicity will be categorised based on the variables available and to allow for harmonisation across the datasets: Asian, Black, Mixed, Other and White. Where data permits, we may use more granular categories of ethnicity. Where numbers are too small and risk identifying individuals, such as in NIMATS, we may collapse the categories to White and Non-white.

(iv) Social deprivation

The patient level Index of Multiple Deprivation specific to each nation will be used and categorised into quintiles.

(v) Body mass index

We shall include the latest available pre-pregnancy body mass index for the pregnant women. Where booking data is available before 16 weeks gestation, this will be used (e.g. in NIMATS). Body mass index will be considered a covariate instead of a health condition. The World Health Organisation's classification of obesity will be used to categorise body mass

For peer review only

index: <18.5 kg/m², 18.5 to 24.9 kg/m², 25.0 to 29.9 kg/m², 30.0 to 34.9 kg/m², 35.0 to 39.9 kg/m², and 40+ kg/m².[35] Categories may be combined where numbers are too small.

(vi) Smoking

We shall include the latest available pre-pregnancy smoking status for the pregnant women. Smoking status will be categorised as: non-smoker, ex-smoker, and smoker.

(vii) Year (pregnancy start date)

Data quality and clinical guidelines may vary by year. Its effect on outcomes will be accounted for by adjusting for year of conception in the analysis.

Statistical analysis

We anticipate analyses will commence in June 2023 with study completion by June 2024. Baseline characteristics of the study population and outcomes will be described with summary statistics. Modified Poisson regression will be performed to estimate the relative risks of study outcomes. Cox regression will be performed for longer-term outcomes. The unit of analysis will be the pregnancy episode.

A federated analysis approach will be used as data governance arrangements do not allow pooling of the data across the four nations. Each dataset will be analysed separately following a common study protocol. A common data model will be established and implemented across the dataset, building on our previous work harmonising the phenome definitions for exposure conditions.[10] The effect sizes will be pooled using random-effects meta-analyses with inverse variance weighting for the primary care and secondary care datasets respectively.[36]

Where rare combinations of health conditions and outcomes may lead to identification of an individual or at the prespecified minimum count allowed by each data source, we will suppress the output.

For peer review only - http://bmjopen.bmj.com/site/about/guidelines.xhtml

Pregnant women with more than one pregnancy episode

An individual may have more than one pregnancy over the study period. The pregnancy episodes of the same woman will not be independent of each other. The severity of the exposure variable (pre-existing multiple long-term conditions) may increase in later pregnancy episodes as the pregnant women accumulates more long-term health conditions. If a woman had an adverse pregnancy outcome, she is more at risk of the same adverse outcome in subsequent pregnancy episodes. We shall account for this clustering effect of women with more than one pregnancy episode during the study period using the Generalised Estimating Equation in the regression analyses.

Multiple pregnancies

The main analysis will be limited to singleton pregnancies. Outcomes for pregnant women with multiple long-term conditions and multiple pregnancies (i.e. twins and higher order pregnancies) will be analysed as a separate cohort.

Missing data

Where exposure and outcome conditions are identified based on diagnostic codes, the absence of the code will be considered as an absence of the condition. The level and types of missingness of covariates will be reviewed and where appropriate will be addressed with representing missing data as a separate category or multiple imputation with chain equation (MICE). For variables required to compute an outcome, missing values will be imputed using MICE. Example of these variables include birthweight, gestational age and baby's sex to

For peer review only - http://bmjopen.bmj.com/site/about/guidelines.xhtml

determine preterm birth and small for gestational age. For each outcome, the statistical analyses will be performed on the imputed datasets and the estimates will be pooled with Rubin's rule.

Sensitivity analyses

We shall conduct sensitivity analyses using (i) complete case analysis, (ii) varying definitions of maternal multiple long-term conditions exposure using D'Arcy et al's core exposure set,[30] and (iii) in primiparous women. The latter is to account for the fact that some long-term conditions can arise from complications from a previous pregnancy.

Patient and public involvement

The research question was informed by discussions with our patient and public involvement (PPI) advisory group and our PPI co-investigators (NM and RP).

The selection of outcomes are guided by our ongoing work developing a core outcome set for studies of pregnant women with multiple long-term conditions, where patients are key stakeholders.[33]

Our PPI advisory group and PPI co-investigators will be involved in interpreting the study findings, producing lay summaries and infographics, and disseminating the study findings through their network.

ETHICS AND DISSEMINATION

Ethics approval

For peer review only - http://bmjopen.bmj.com/site/about/guidelines.xhtml

CPRD: CPRD has broad National Research Ethics Service Committee ethics approval for purely observational research using the primary care data and established data linkages. The study has been reviewed and approved by CPRD's Independent Scientific Advisory Committee (reference: 20_181R).

SAIL: In accordance with UK Health Research Authority guidance, ethical approval is not mandatory for studies using only anonymised data. The study has been approved by SAIL Information Governance Review Panel.

Scotland dataset: The study has been approved by the National Health Service Scotland Public Benefit and Privacy Panel for Health and Social Care (HSC-PBPP) and The University Teaching and Research Ethics Committee (UTREC) from the University of St Andrews.

NIMATS: The study has been approved by the Honest Broker Service Governance Board.

Born in Bradford: Ethics approval was granted by Bradford National Health Service Research Ethics Committee (ref 07/H1302/112) for the Born in Bradford cohort.

The proposed study is purely observational and will use anonymised research data. The study will not involve participant recruitment. Therefore, consent to participate is not required.

Consent for publication

This is not applicable as the manuscript is a study protocol. In the proposed study, we will use de-identified study data, therefore consent for publication will not be required.

Dissemination

Study findings will be submitted for publications in peer reviewed journals and presented at key conferences for health and social care professionals involved in the care of pregnant women with multiple long-term conditions and their children. We will also organise

For peer review only - http://bmjopen.bmj.com/site/about/guidelines.xhtml

dissemination events to share our findings with the public, service users, clinicians and researchers.

DISCUSSION

MuM-PreDiCT is a consortium across all four nations of the UK studying multiple long-term conditions in pregnancy. As part of MuM-PreDiCT's program of work, we outlined the protocol for an observational study of maternal and child outcomes for pregnant women with multiple long-term conditions, using routine health records and a birth cohort in the UK.

Comparison with current literature

A recent systematic review found seven observational studies on the association of pre-pregnancy multiple long-term conditions with adverse maternal outcomes.[8] The review found that pre-pregnancy multiple long-term conditions were associated with severe maternal morbidity, hypertensive disorders of pregnancy, and acute health care use in the perinatal period.[8] Most studies were conducted in the United States.[8] Authors of the review commented that many studies included conditions arising in pregnancy in defining multiple long-term conditions, making it difficult to examine the impact of chronic conditions on maternal health.[8]

This proposed study will be based in the UK and will use a broad range of long-term conditions selected by women and clinicians to define multiple long-term conditions. Pregnancy related conditions and complications will be treated as study outcomes and will not be included in the exposure's definition. We will also study outcomes across all stages of pregnancy and outcomes for both women and their children.

For peer review only - http://bmjopen.bmj.com/site/about/guidelines.xhtml

For peer review only

Strengths and limitations

This proposed study will utilise routine health records from all four nations of the UK (England, Scotland, Wales and Northern Ireland). The available data sources consist of anonymised patient records from primary and secondary care, community prescription data, and maternity care data from routine booking appointments (first antenatal appointment offered universally and as the gateway to access maternity care in the UK).

Rich data will also be available from a birth cohort from Bradford, an ethnically diverse population in England. Beyond examining maternal outcomes, linked mother baby data and the birth cohort data will allow for the exploration of child outcomes. The key strength of this proposed study therefore is the generalisability of study findings to the UK population. Observing similar effect sizes across the different datasets will also increase the confidence in the study findings. Conversely, discrepancy in findings will stimulate further exploration of the datasets which may generate new knowledge.

As this is an observational study using anonymised routine health records, key limitations include missing data, misclassification bias due to inaccurate clinical coding and residual confounding.

Maternal multimorbidity will be quantified with simple counts. A systematic review of comorbidity indices used in maternal health research found three indices: Maternal Comorbidity Index, Charlson comorbidity index and Elixhauser comorbidity index.[27] Only the Maternal Comorbidity Index was developed from pregnant and postpartum women.[27] It was developed using hospital data with 20 maternal comorbidities but it included pregnancy related complications and factors such as multiple gestation, gestational diabetes, and hypertension disorder of pregnancy.[27 28] In contrast, the list of health conditions we will use

For peer review only - http://bmjopen.bmj.com/site/about/guidelines.xhtml

to define maternal pre-existing multimorbidity is more comprehensive and included leading causes of indirect maternal death (e.g. epilepsy) and mental health conditions.

Nevertheless, when using simple counts to quantify multiple long-term conditions, the severity of each health conditions will not be captured. The dose-response relationship will only be reflected in the total number of pre-existing long-term conditions. For example, we will not be able to distinguish the outcomes for a pregnant woman with diet controlled diabetes and mild asthma from a pregnant woman with insulin dependent diabetes and brittle asthma. However, pregnant women with severe conditions are more likely to receive intense specialist care than pregnant women with mild conditions. As the number of pregnant women with greater disease severity is likely to be smaller than those with milder condition, adverse pregnancy outcomes may be underestimated.

Exposure and outcome events are only captured in routine health records when the pregnant women have presented to primary or secondary care and therefore the true prevalence and incidence may be underestimated. Health conditions that are managed conservatively in primary care, such as depression, anxiety and miscarriage, may not be captured in secondary care datasets. Events such as termination of pregnancy that occurred outside of the traditional health care settings may also be underestimated.[37] Similarly, antenatal hospital admission data may not reflect the full burden of additional antenatal appointments or acute care attendances, as care accessed through other routes may not be captured.

Body mass index, which encompasses underweight and obese categories, will be studied as a covariate instead of being counted as part of multimorbidity. There is much debate around whether obesity should be considered a disease[38] or a risk factor for other long-term conditions such as cardiometabolic conditions and cancers.[39-41] What is clear is pre-

For peer review only - http://bmjopen.bmj.com/site/about/guidelines.xhtml

pregnancy maternal obesity is associated with adverse pregnancy outcome and dedicated care guideline has been established to manage this risk.[42 43]

Clinical implications

Current obstetric guidelines for pregnant women with medical conditions are focused on specific and single health conditions. There are currently no guidelines for the management of pregnant women with multiple long-term conditions in the UK. The heterogeneity of multiple long-term conditions means an all-encompassing guideline for every possible combination of long-term conditions would not be possible. Indeed the English national guideline for multimorbidity focuses on general approaches such as coordinated and holistic care, improving quality of life by reducing treatment burden and shared decision making between patients and clinicians.[44] A guideline for multiple long-term conditions (multimorbidity) in pregnancy is likely to follow the same principles but with additional focus on the maternity care aspect.

The basis of shared decision making is the provision of evidence based information. As observed in the systematic review, there is currently a lack of evidence on the consequences of pregnancy for women with multiple long-term conditions.[8] Our PPI advisory group and preliminary findings from our core outcome set development work have highlighted how women valued having information to help them mentally prepare to face potential adverse pregnancy outcomes. The output from this study will therefore provide valuable information for women to make informed decision with their clinicians about family planning and their preconception, pregnancy and postpartum care. It will also provide valuable information to guide the future design of care pathway for women with multiple long-term conditions.

For peer review only - http://bmjopen.bmj.com/site/about/guidelines.xhtml

For peer review only

For peer review only - http://bmjopen.bmj.com/site/about/guidelines.xhtml

ABBREVIATIONS

CPRD	Clinical Practice Research Datalink
ICD-10	International Classification of Disease 10th version
MICE	Multiple imputation with chain equation
NICE	National Institute for Health and Care Excellence
NIMATS	Northern Ireland Maternity System
OPCS	Operating Procedures Codes
PPI	Patient and public involvement
SAIL	Secure Anonymised Information Linkage
UK	United Kingdom

For peer review only

For peer review only - http://bmjopen.bmj.com/site/about/guidelines.xhtml

Data availability statement

Not applicable for the present protocol manuscript. In the proposed study, the data that support the findings are available from CPRD, SAIL, Scotland National Health Service Scotland Public Benefit and Privacy Panel for Health and Social Care, NIMATS and Born in Bradford, but restrictions apply to the availability of these data, which are used under license for the current study, and so are not publicly available. However, data will be available from the authors upon reasonable request and with permission of CPRD, SAIL, Scotland National Health Service Scotland Public Benefit and Privacy Panel for Health and Social Care, NIMATS and Born in Bradford.

Competing interests

The authors declare that they have no competing interests.

Funding

This work is funded by the Strategic Priority Fund "Tackling multimorbidity at scale" programme (grant number MR/W014432/1) delivered by the Medical Research Council and the National Institute for Health Research (NIHR) in partnership with the Economic and Social Research Council and in collaboration with the Engineering and Physical Sciences Research Council. The views expressed are those of the author and not necessarily those of the funders, the NIHR or the UK Department of Health and Social Care. The funders had no role in study design, decision to publish, or preparation of the manuscript.

Contributors

SIL: conceptualisation, funding acquisition, methodology, and writing (original draft preparation). KN, MB, KAE, KMA, and DOR: conceptualisation, funding acquisition, methodology, supervision, and writing (review and editing). HH, GS, AS, NM, AAL, AFF, CNP, CY, CMC, JIK, PB, RP, RR, ST, SB, UA, and ZV: conceptualisation, funding acquisition, methodology, and writing (review and editing). LK, KP, MS, MM, NC, and

For peer review only

For peer review only - http://bmjopen.bmj.com/site/about/guidelines.xhtml

SPBHS: conceptualisation, methodology, and writing (review and editing). MuM-PreDiCT group: conceptualisation, funding acquisition. All authors read and approved the manuscript.

For peer review only

For peer review only - http://bmjopen.bmj.com/site/about/guidelines.xhtml

REFERENCES

1. Eggleton EJ, McMurrugh KJ, Aiken CE. Maternal pregnancy outcomes in women with cardiomyopathy: a systematic review and meta-analysis. *Am J Obstet Gynecol* 2022;227(4):582-92. doi: 10.1016/j.ajog.2022.05.039 [published Online First: 20220521]
2. Roos-Hesselink J, Baris L, Johnson M, et al. Pregnancy outcomes in women with cardiovascular disease: evolving trends over 10 years in the ESC Registry Of Pregnancy And Cardiac disease (ROPAC). *Eur Heart J* 2019;40(47):3848-55. doi: 10.1093/eurheartj/ehz136
3. Wiles K, Webster P, Seed PT, et al. The impact of chronic kidney disease Stages 3–5 on pregnancy outcomes. *Nephrology Dialysis Transplantation* 2020;36(11):2008-17. doi: 10.1093/ndt/gfaa247
4. Viale L, Allotey J, Cheong-See F, et al. Epilepsy in pregnancy and reproductive outcomes: a systematic review and meta-analysis. *Lancet (London, England)* 2015;386(10006):1845-52. doi: 10.1016/s0140-6736(15)00045-8 [published Online First: 20150825]
5. World Health Organization. Multimorbidity: Technical Series on Safer Primary Care. 2016. https://apps.who.int/iris/bitstream/handle/10665/252275/9789241511650-eng.pdf.
6. Admon LK, Winkelman TNA, Heisler M, et al. Obstetric Outcomes and Delivery-Related Health Care Utilization and Costs Among Pregnant Women With Multiple Chronic Conditions. *Preventing Chronic Disease* 2018;15:E21.
7. D'Arcy R, Knight M, Mackillop L. A retrospective audit of the socio-demographic characteristics and pregnancy outcomes for all women with multiple medical problems giving birth at a tertiary hospital in the UK in 2016. *BJOG: An International Journal of Obstetrics and Gynaecology* 2019;126:128.
8. Brown HK, McKnight A, Aker A. Association between pre-pregnancy multimorbidity and adverse maternal outcomes: A systematic review. *Journal of multimorbidity and comorbidity* 2022;12:26335565221096584. doi: 10.1177/26335565221096584 [published Online First: 2022/05/20]
9. Knight M, Bunch K, Tuffnell D, et al. Saving Lives, Improving Mothers' Care - Lessons learned to inform maternity care from the UK and Ireland Confidential Enquiries into Maternal Deaths and Morbidity 2016-18. Oxford: National Perinatal Epidemiology Unit, University of Oxford, 2020.
10. Lee SI, Azcoaga-Lorenzo A, Agrawal U, et al. Epidemiology of pre-existing multimorbidity in pregnant women in the UK in 2018: a population-based cross-sectional study. *BMC pregnancy and childbirth* 2022;22(1):120. doi: 10.1186/s12884-022-04442-3
11. Office for National Statistics. Childbearing for women born in different years, England and Wales: 2020. 2022 [cited 2022 20th December]. Available from: https://www.ons.gov.uk/peoplepopulationandcommunity/birthsdeathsandmarriages/conceptionandfertilityrates/bulletins/childbearingforwomenbornindifferentyearsenglandandwales/2020.
12. OECD Family Database. SF2.3: Age of mothers at childbirth and age-specific fertility. 2022. https://www.oecd.org/els/soc/SF_2_3_Age_mothers_childbirth.pdf.
13. Chen C, Xu X, Yan Y. Estimated global overweight and obesity burden in pregnant women based on panel data model. *PLoS One* 2018;13(8):e0202183. doi: 10.1371/journal.pone.0202183 [published Online First: 20180809]
14. Wang MC, Freaney PM, Perak AM, et al. Trends in Prepregnancy Obesity and Association With Adverse Pregnancy Outcomes in the United States, 2013 to 2018. *J Am Heart Assoc* 2021;10(17):e020717. doi: 10.1161/jaha.120.020717 [published Online First: 20210825]
15. Beeson JG, Homer CSE, Morgan C, et al. Multiple morbidities in pregnancy: Time for research, innovation, and action. *PLoS medicine* 2018;15(9):e1002665. doi: 10.1371/journal.pmed.1002665 [published Online First: 2018/09/27]

For peer review only

For peer review only - http://bmjopen.bmj.com/site/about/guidelines.xhtml

16. Herrett E, Gallagher AM, Bhaskaran K, et al. Data Resource Profile: Clinical Practice Research Datalink (CPRD). *International Journal of Epidemiology* 2015;44(3):827-36. doi: 10.1093/ije/dyv098
17. National Institute for Health and Care Excellence. Clinical Knowledge Summaries: Antenatal Care - Uncomplicated Pregnancy. 2021 [Available from: https://cks.nice.org.uk/topics/antenatal-care-uncomplicated-pregnancy/management/antenatal-care-uncomplicated-pregnancy/#booking-appointment accessed 5th July 2022.
18. Hatherall B, Morris J, Jamal F, et al. Timing of the initiation of antenatal care: An exploratory qualitative study of women and service providers in East London. *Midwifery* 2016;36:1-7. doi: 10.1016/j.midw.2016.02.017 [published Online First: 2016/04/24]
19. Office for National Statistics. Birth characteristics in England and Wales: 2020. 2022 [Available from: https://www.ons.gov.uk/peoplepopulationandcommunity/birthsdeathsandmarriages/livebirths/bulletins/birthcharacteristicsinenglandandwales/2020#place-of-birth accessed 12th July 2022.
20. Raynor P. Born in Bradford, a cohort study of babies born in Bradford, and their parents: protocol for the recruitment phase. *BMC public health* 2008;8:327. doi: 10.1186/1471-2458-8-327 [published Online First: 2008/09/25]
21. CPRD. Release notes: CPRD GOLD May 2022. 2022 [Available from: https://cprd.com/sites/default/files/2022-05/2022-05%20CPRD%20GOLD%20Release%20Notes.pdf accessed 14th July 2022.
22. SAIL Databank. 10 years of spearheading data privacy and research utility [cited 2021 16th February]. Available from: https://saildatabank.com/wp-content/uploads/SAIL_10_year_anniversary_brochure.pdf.
23. Health Data Research Innovation Gateway. Northern Ireland Regional Maternity system (NIMATS) 2021 [cited 2021 7 December]. Available from: https://web.www.healthdatagateway.org/dataset/89d76819-32cb-48ad-9337-880f324a6ec0.
24. Born in Bradford. About Us 2021 [cited 2021 7 December]. Available from: https://borninbradford.nhs.uk/about-us/.
25. Ho IS, Azcoaga-Lorenzo A, Akbari A, et al. Variation in the estimated prevalence of multimorbidity: systematic review and meta-analysis of 193 international studies. *BMJ Open* 2022;12(4):e057017. doi: 10.1136/bmjopen-2021-057017 [published Online First: 20220429]
26. Ho IS-S, Azcoaga-Lorenzo A, Akbari A, et al. Examining variation in the measurement of multimorbidity in research: a systematic review of 566 studies. *The Lancet Public Health* 2021;6(8):e587-e97. doi: 10.1016/S2468-2667(21)00107-9
27. Aoyama K, D'Souza R, Inada E, et al. Measurement properties of comorbidity indices in maternal health research: a systematic review. *BMC Pregnancy & Childbirth* 2017;17(1):372.
28. Bateman BT, Mhyre JM, Hernandez-Diaz S, et al. Development of a comorbidity index for use in obstetric patients. *Obstetrics and Gynecology* 2013;122(5):957-65. doi: 10.1097/AOG.0b013e3182a603bb
29. Kuan V, Denaxas S, Gonzalez-Izquierdo A, et al. A chronological map of 308 physical and mental health conditions from 4 million individuals in the English National Health Service. *Lancet Digit Health* 2019;1(2):e63-e77. doi: 10.1016/s2589-7500(19)30012-3 [published Online First: 20190520]
30. D'Arcy R, Knight M. List of health conditions to define multiple long-term conditions in pregnancy. [Available from: https://drive.google.com/file/d/1Q3LTzcXGnEFcS6eMAA0pntEUUUa-iTtp/view accessed 14th July 2022.

For peer review only - http://bmjopen.bmj.com/site/about/guidelines.xhtml

31. Devane D, Begley CM, Clarke M, et al. Evaluating maternity care: a core set of outcome measures. *Birth (Berkeley, Calif)* 2007;34(2):164-72. doi: 10.1111/j.1523-536X.2006.00145.x [published Online First: 2007/06/05]
32. Nijagal MA, Wissig S, Stowell C, et al. Standardized outcome measures for pregnancy and childbirth, an ICHOM proposal. *BMC Health Services Research* 2018;18(1):953. doi: 10.1186/s12913-018-3732-3
33. Lee SI, Eastwood K-A, Moss N, et al. Protocol for the development of a core outcome set for studies of pregnant women with pre-existing multimorbidity. *BMJ Open* 2021;11(10):e044919. doi: 10.1136/bmjopen-2020-044919
34. World Health Organization. WHO Technical Consultation on Postpartum and Postnatal Care,. 2010. https://www.ncbi.nlm.nih.gov/books/NBK310595/ (accessed 6th July 2022).
35. World Health Organization. Obesity: preventing and managing the global epidemic. Report of a WHO consultation. *World Health Organization technical report series* 2000;894:i-xii, 1-253. [published Online First: 2001/03/10]
36. Borenstein M, Hedges LV, Higgins JP, et al. A basic introduction to fixed-effect and random-effects models for meta-analysis. *Res Synth Methods* 2010;1(2):97-111. doi: 10.1002/jrsm.12 [published Online First: 20101121]
37. Minassian C, Williams R, Meeraus WH, et al. Methods to generate and validate a Pregnancy Register in the UK Clinical Practice Research Datalink primary care database. *Pharmacoepidemiology and Drug Safety* 2019;28(7):923-33. doi: https://doi.org/10.1002/pds.4811
38. Burki T. European Commission classifies obesity as a chronic disease. *The Lancet Diabetes & Endocrinology* 2021;9(7):418. doi: 10.1016/S2213-8587(21)00145-5
39. Abdullah A, Peeters A, de Courten M, et al. The magnitude of association between overweight and obesity and the risk of diabetes: a meta-analysis of prospective cohort studies. *Diabetes Res Clin Pract* 2010;89(3):309-19. doi: 10.1016/j.diabres.2010.04.012 [published Online First: 20100520]
40. Bhaskaran K, Douglas I, Forbes H, et al. Body-mass index and risk of 22 specific cancers: a population-based cohort study of 5.24 million UK adults. *The Lancet* 2014;384(9945):755-65. doi: 10.1016/S0140-6736(14)60892-8
41. Powell-Wiley TM, Poirier P, Burke LE, et al. Obesity and Cardiovascular Disease: A Scientific Statement From the American Heart Association. *Circulation* 2021;143(21):e984-e1010. doi: doi:10.1161/CIR.0000000000000973
42. D'Souza R, Horyn I, Pavalagantharajah S, et al. Maternal body mass index and pregnancy outcomes: a systematic review and metaanalysis. *American Journal of Obstetrics & Gynecology MFM* 2019;1(4) doi: 10.1016/j.ajogmf.2019.100041
43. Royal College of Obstetricians & Gynaecologists. Care of Women with Obesity in Pregnancy (Green-top Guideline No. 72) 2018 [cited 2023 3rd January]. Available from: https://www.rcog.org.uk/guidance/browse-all-guidance/green-top-guidelines/care-of-women-with-obesity-in-pregnancy-green-top-guideline-no-72/.
44. National Institute for Health and Care Excellence. NICE guideline [NG56]: Multimorbidity: clinical assessment and management. 2016. https://www.nice.org.uk/guidance/ng56/resources/multimorbidity-clinical-assessment-and-management-pdf-1837516654789.

For peer review only - http://bmjopen.bmj.com/site/about/guidelines.xhtml
